# Study on Damage Evolution and Mechanical Performance of PCCP Before and After Internal Steel-Cylinder Repair Under Multiple Broken-Wire Conditions

**DOI:** 10.3390/ma19163414

**Published:** 2026-08-11

**Authors:** Jinyan Si, Guangming Wang, Yifan Zheng, Junxiao He, Ruihang Wang

**Affiliations:** 1 Beijing Municipal Engineering Research Institute, Beijing 100037, China; 18610286632@163.com (J.S.); northlemontree@163.com (G.W.); 2 School of Civil and Transportation Engineering, Beijing University of Civil Engineering and Architecture, Beijing 100044, China; 2108140224002@stu.bucea.edu.cn (Y.Z.); 2108140224015@stu.bucea.edu.cn (R.W.)

**Keywords:** prestressed concrete cylinder pipe, broken-wire damage, internal steel-cylinder repair, multiple broken-wire conditions, full-scale model test, finite element analysis

## Abstract

To evaluate the damage evolution of prestressed concrete cylinder pipe (PCCP) with broken wires and the effectiveness of internal steel-cylinder repair, a three-dimensional finite element model was established using ABAQUS and calibrated with a full-scale hydrostatic-pressure test. Test-calibrated parametric simulations considered three nominal wire breakage levels (5%, 15%, and 25%) and two damage locations: the pipe midspan and pipe-end socket region. Concrete-core damage, original steel-cylinder strain, strengthening-cylinder response, and grouting-layer load transfer were compared before and after repair. The unrepaired pipe-end socket region showed a lower numerical nonlinear-transition pressure and stronger damage propagation because of geometric discontinuity. Increasing wire breakage reduced the nonlinear-transition pressure of the concrete core and increased the original steel-cylinder strain. After internal steel-cylinder repair, the structural responses converged across the investigated damage cases. At 1.2 MPa, the calculated strain reductions between corresponding unrepaired and repaired numerical configurations were 77.2–91.0% for the original steel cylinder. At the maximum applied pressure of 1.6 MPa, the modeled maximum original steel-cylinder strain was 459.11 με. The repaired configurations exhibited reduced strain demand and coordinated load sharing over the investigated monotonic internal-pressure range. The test program ended at 1.6 MPa without an ultimate-failure loading stage.

## 1. Introduction

Prestressed concrete cylinder pipe (PCCP), composed of a concrete core, thin steel cylinder, prestressing wires, and mortar coating, is widely used in large-diameter, long-distance, and high-pressure water conveyance projects [1]. During service, corrosion, groundwater attack, abnormal cathodic protection, construction deviations, material defects, and load fluctuations may degrade the prestressing system and cause wire breakage [2]. The resulting loss of circumferential precompression can trigger mortar-coating cracking, concrete-core damage, yielding of the original steel cylinder, or even pipe bursting [3,4]. Reliable assessment of broken-wire PCCP and repair methods capable of restoring stiffness and load-transfer capacity are therefore essential for the safe operation of water-conveyance lifelines [1].

Existing studies have clarified important aspects of broken-wire damage in PCCP. Theoretical and numerical models have been used to analyze the basic mechanical response under combined loads, longitudinal cracking, original steel-cylinder yielding, joint interaction, mortar-coating bond quality, and clustered wire fracture [5,6,7,8,9,10,11,12,13]. Full-scale tests and monitoring studies have further revealed interlayer strain development, failure modes, and continuous strain responses during internal-pressure loading and wire breakage [14,15,16,17]. Sun et al. [18] also examined the influence of different wire breakage ratios on internal and external pressure capacity. These studies provide the basis for evaluating damage evolution and mechanical-response degradation in PCCP with broken wires.

Repair technologies for damaged PCCP mainly include fiber-reinforced composite linings, external prestressing, and steel structural strengthening. FRP or CFRP linings can delay concrete cracking and restore part of the circumferential restraint [19,20,21,22,23], while external prestressed steel strands or prestressed CFRP can compensate for prestress loss and improve the stress state of pipes with broken wires [24,25]. However, when wire breakage is extensive and pipe-wall damage is severe, flexible strengthening materials may have limited ability to reconstruct global stiffness. Rigid internal steel-cylinder repair, such as steel-pipe sliplining or necked steel-cylinder lining, adds a new internal bearing layer and forms a composite system consisting of the strengthening steel cylinder, grouting layer, and original pipe core. In this system, the radial compression transfer and deformation-coordination capacity of the grouting layer are critical to load redistribution [26,27].

Previous studies have established methods for broken-wire assessment and numerical evaluation of damaged PCCP, while separate investigations have examined repair and strengthening technologies. Direct comparisons of internal steel-cylinder repair configurations across different clustered broken-wire levels and representative pipe locations, together with component-level evaluation of the original and strengthening systems, remain limited.

This study combines a full-scale hydrostatic-pressure test with a three-dimensional finite-element framework. The numerical investigation considers nominal broken-wire levels of 5%, 15%, and 25% at the pipe midspan and pipe-end socket region. Concrete-core damage, original steel-cylinder strain, strengthening-cylinder response, and grouting-layer radial load transfer are compared within the investigated monotonic internal-pressure range. The contribution lies in the integrated comparison of damage level, damage location, original-pipe response, and internal strengthening response within a unified experimental–numerical framework.

## 2. Test Program

The full-scale hydrostatic-pressure test was used to obtain strain responses and crack-development phenomena before and after internal steel-cylinder repair. These observations provided the loading procedure, boundary reference, and experimental benchmark for finite element calibration.

### 2.1. Specimen Design and Material Properties

**Figure 1:**
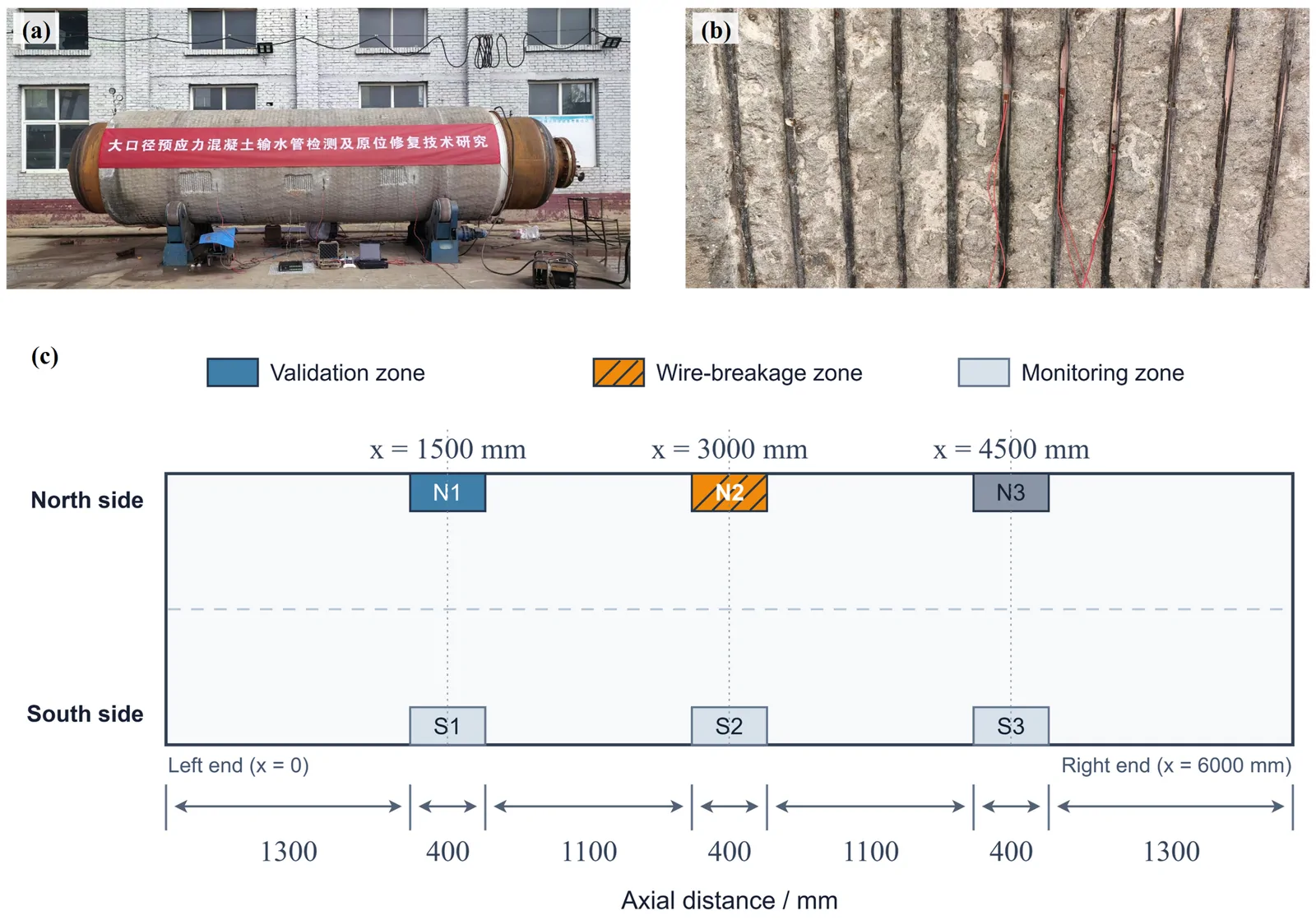
Full-scale test specimen and strain-measurement arrangement: (**a**) full-scale PCCP specimen; (**b**) strain gauges installed on adjacent exposed prestressing wires; (**c**) axial distribution and functional classification of the measurement windows. The Chinese text on the banner in panel (**a**) means “Research on inspection and in situ repair technology for large-diameter prestressed concrete water-conveyance pipes”.

The prototype pipe was a PCCP-E pipe used in an actual water-diversion project. As shown in Figure 1, the pipe length was 6000 mm and the inner diameter was 1600 mm. The total thickness of the concrete core was 110 mm. A 1.5-mm-thick original impervious steel cylinder, hereafter referred to as the original steel cylinder, was located in the middle of the wall and divided the core into a 50-mm-thick inner concrete layer and a 58.5-mm-thick outer concrete layer. The prestressing wires were 5.0-mm-diameter high-strength wires wound on the outer-core surface at a spacing of 35.5 mm, with a standard tensile strength of 1540 MPa. The outermost layer was a 45-mm-thick cement mortar coating. The physical and mechanical parameters of the concrete, steel, and mortar were provided by the manufacturer, and the elastic parameters were used to define the linear-elastic stage of the finite element model.

### 2.2. Internal Strengthening Steel-Cylinder Repair Configuration

**Figure 2:**
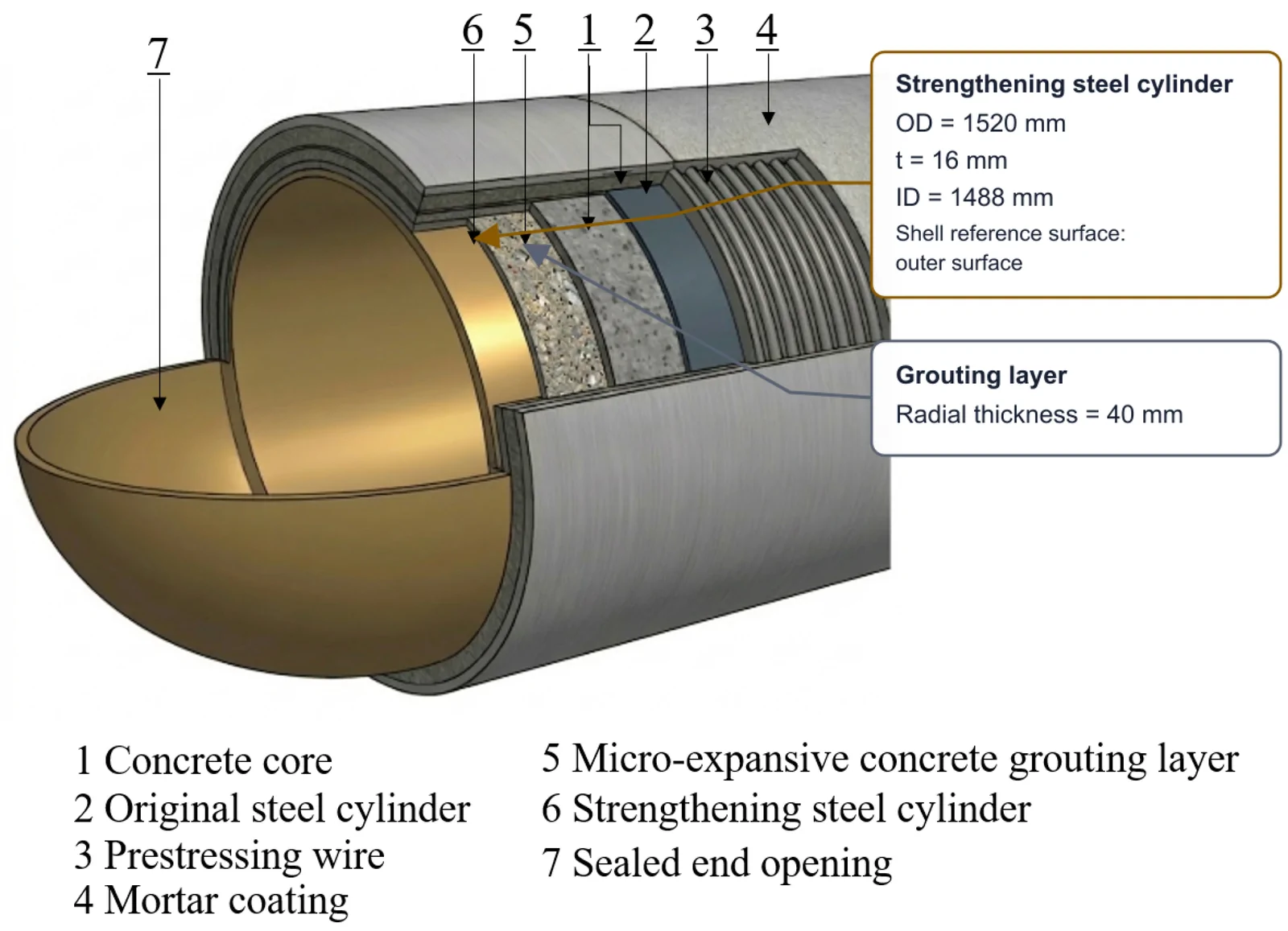
Schematic of the PCCP repair configuration and the principal dimensions of the strengthening steel cylinder and grouting layer.

For PCCP with broken-wire damage, the test adopted a composite repair process combining an internal strengthening steel cylinder and micro-expansive concrete grouting. As shown in Figure 2, a Q345 strengthening steel cylinder was installed inside the original pipe. The strengthening steel cylinder had an outer diameter of 1520 mm, an inner diameter of 1488 mm, and a wall thickness of 16 mm. Because the inner diameter of the original concrete core was 1600 mm, the radial clearance between the outer surface of the strengthening cylinder and the inner surface of the original concrete core was 40 mm, forming the annular grouting layer. The strengthening cylinder was welded and sealed to the original pipe-end steel components using annular steel plates at both ends, and micro-expansive concrete was injected into the closed annular space. After hardening, the grouting layer filled the annular space and transferred radial compression between the strengthening cylinder and the original pipe core, enabling the repaired components to share load under internal pressure.

### 2.3. Loading Program, Measurement Layout, and Broken-Wire Simulation

The full-scale test comprised four sequential stages: hydrostatic loading of the original pipe before artificial wire cutting, local cutting of approximately 5% of the prestressing wires, installation of the internal strengthening steel cylinder and grouting of the annular space, and hydrostatic loading of the repaired pipe. Six strain-measurement windows were arranged on the north and south sides of the pipe at axial center positions of x=1500, 3000, and 4500 mm. Each window covered an axial length of 400 mm and a circumferential extent of 300 mm. Strain gauges were installed on the prestressing wires, mortar coating, and concrete core after local removal of the mortar coating. The measurements used in the present comparison were oriented in the circumferential direction. Within each prestressing-wire measurement window, gauges were arranged on adjacent exposed wires. The N1, N2, and N3 windows represented the validation, controlled wire-breakage, and monitoring zones, respectively, with S1–S3 located at the corresponding axial positions on the south side.

**Figure 3:**
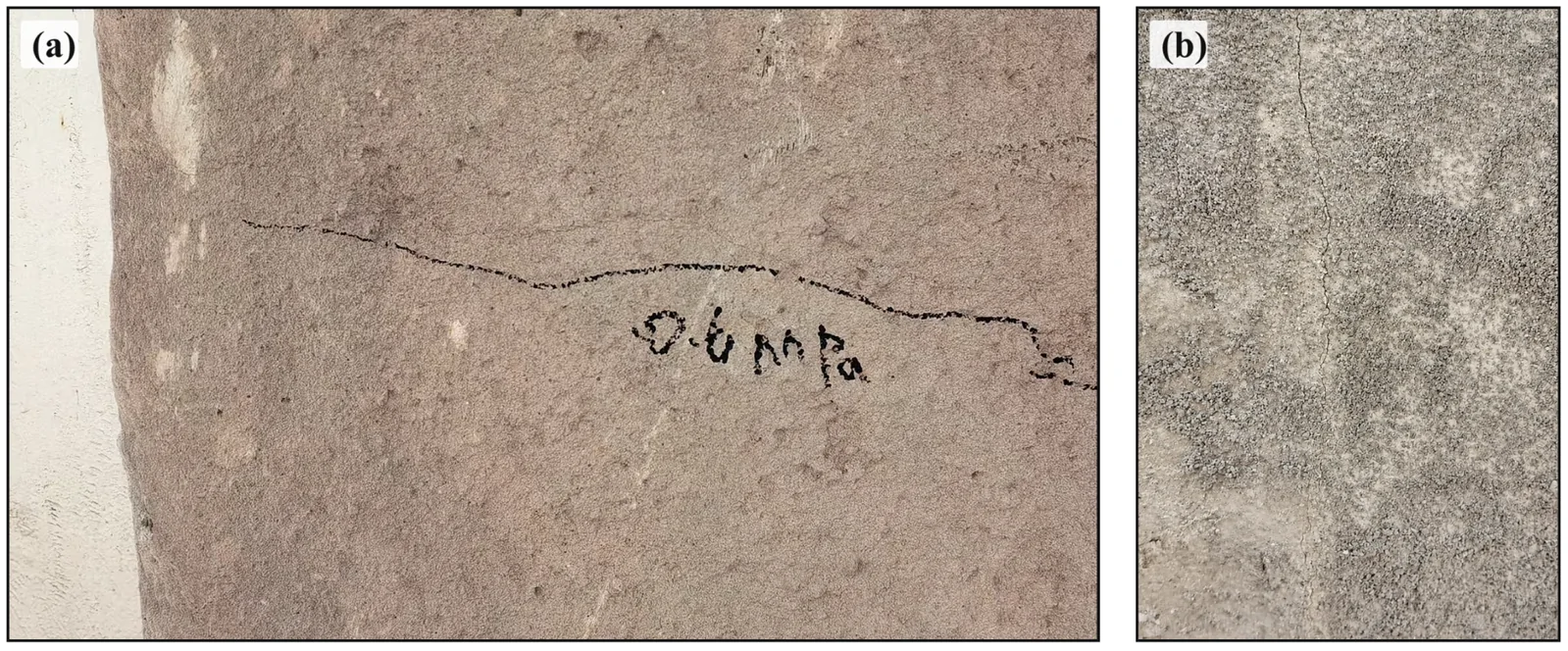
Representative surface cracks observed during hydrostatic loading of the original pipe: (**a**) circumferential crack recorded near 0.6 MPa at the pipe-end socket region; (**b**) representative axial crack recorded during subsequent loading.

In the first stage, internal water pressure was applied to the original pipe in increments of 0.1 MPa. The holding time was 2 min at each pressure level from 0.1 to 0.3 MPa, 3 min from 0.4 to 0.6 MPa, 4 min from 0.7 to 0.9 MPa, and 5 min from 1.0 to 1.2 MPa. This pressure-dependent schedule was prescribed before loading, with longer holding periods at higher pressure levels providing additional time for inspection and data recording. During the holding-stage inspection, the first surface-visible circumferential crack was identified at the pipe-end socket region when the internal pressure reached approximately 0.6 MPa, as shown in Figure 3a. A representative axial crack recorded during subsequent loading is shown in Figure 3b. At 1.2 MPa, the previously observed surface cracks continued to extend, after which the original-pipe loading stage was terminated. The reported cracking pressure of approximately 0.6 MPa is subject to the 0.1 MPa loading-step resolution.

After the first stage, several prestressing wires were cut using a grinder in the N2 measurement region, forming an approximately 5% local wire breakage level. The strengthening steel cylinder was then installed, and micro-expansive concrete was grouted into the annular space between the strengthening cylinder and the original pipe core. After the grouting layer hardened, post-repair loading used the same 0.1 MPa pressure increment and holding-time schedule up to 1.2 MPa. Each subsequent pressure level from 1.3 to 1.6 MPa was held for 5 min. During post-repair loading to 1.6 MPa, no new through cracks or macroscopic instability was observed, and the measured component strains generally increased with water pressure.

The full-scale test provided the loading program, measured responses, and observed failure phenomena used for model calibration. Hydrostatic-pressure tests were conducted for two structural states: the original pipe before artificial wire cutting and the repaired pipe after approximately 5% local wire cutting. No hydrostatic-pressure test was conducted on the unrepaired pipe after wire cutting. The unrepaired 5% configuration and all 15% and 25% configurations were numerical parametric extensions based on the calibrated model. The unrepaired and repaired numerical configurations were initialized independently; the analysis did not simulate a continuous sequence of pressure-induced damage, repair installation, and reloading.

## 3. Finite Element Model and Validation

Based on the full-scale test, separate three-dimensional finite-element configurations were established for the unrepaired and repaired PCCP using ABAQUS 2024. Both configurations adopted unified material definitions, boundary conditions, prestress conditions, and loading procedures. The repaired configuration incorporated the corresponding nominal broken-wire level, strengthening steel cylinder, and grouting layer. The first-stage measured strains and observed response were used for model calibration and definition of the comparative analysis conditions. This procedure provides a consistent numerical basis for comparing the unrepaired and repaired configurations under monotonic internal pressure.

### 3.1. Material Constitutive Models

The material definitions were standardized using the repaired-model input as the common numerical baseline. The pipe-core concrete and grouting layer were assigned tabulated Concrete Damaged Plasticity (CDP) relations. The steel cylinders were assigned elastic–plastic constitutive relations. Table 1 lists the elastic properties of the unified baseline.

**Figure 4:**
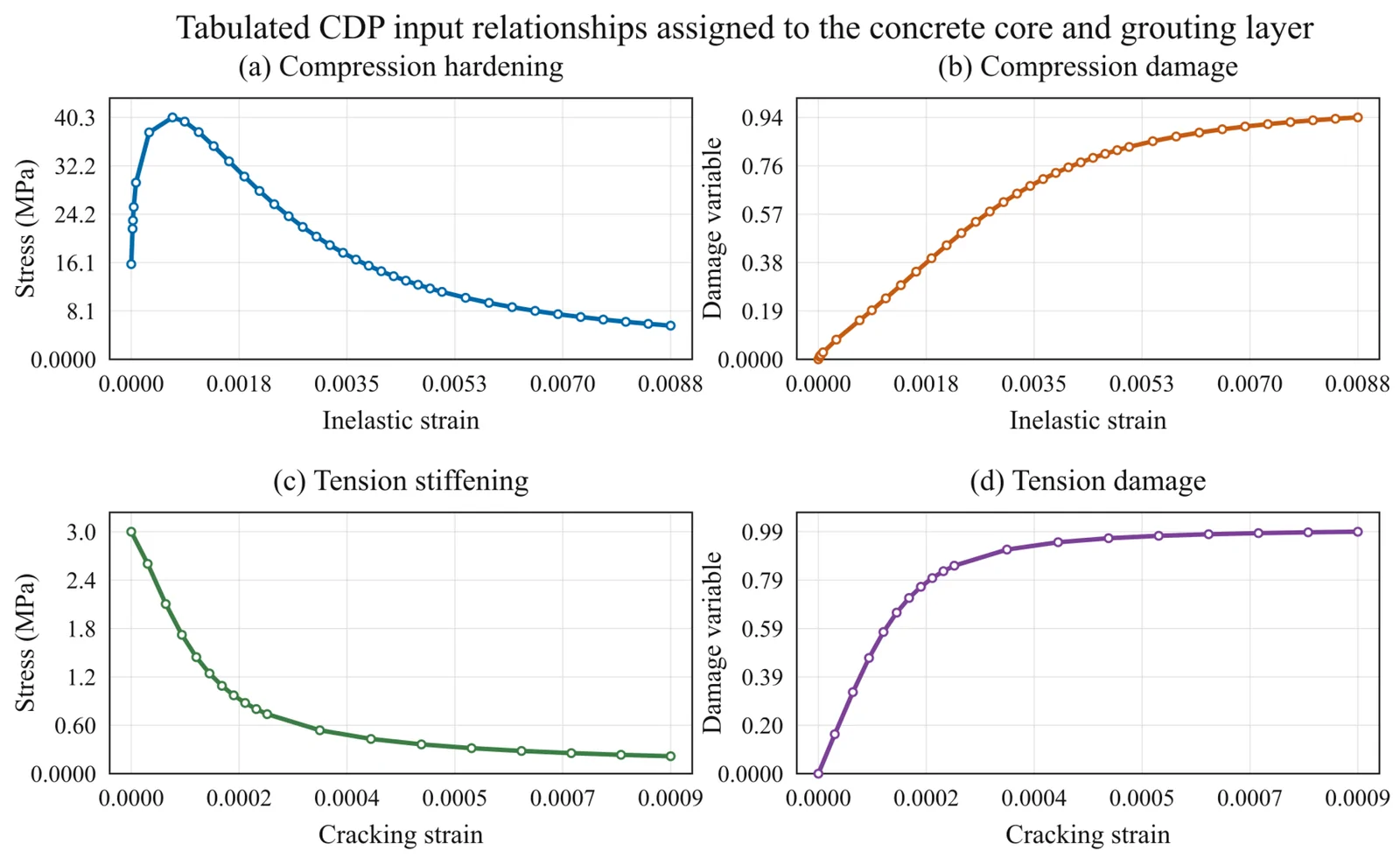
Tabulated CDP input relationships assigned to the pipe-core concrete and grouting layer: (**a**) compression hardening; (**b**) compression damage; (**c**) tension stiffening; and (**d**) tension damage.

The pipe-core and grouting materials were assigned the same tabulated CDP relation in the adopted numerical model. The CDP parameters were a dilation angle of $\psi =30^{\circ }$, eccentricity of ϵ=0.10, $f_{b0}/f_{c0}=1.333$, $K_{c}=0.6667$, and viscosity parameter μ=0.001, as summarized in Table 2. The complete compression-hardening, tension-stiffening, compression-damage, and tension-damage inputs are plotted in Figure 4. Tensile softening was entered as stress versus cracking strain. Fracture-energy regularization and explicit characteristic-length correction were not applied; the viscosity parameter was used for numerical convergence and was not treated as a mesh-objectivity regularization.

**Figure 5:**
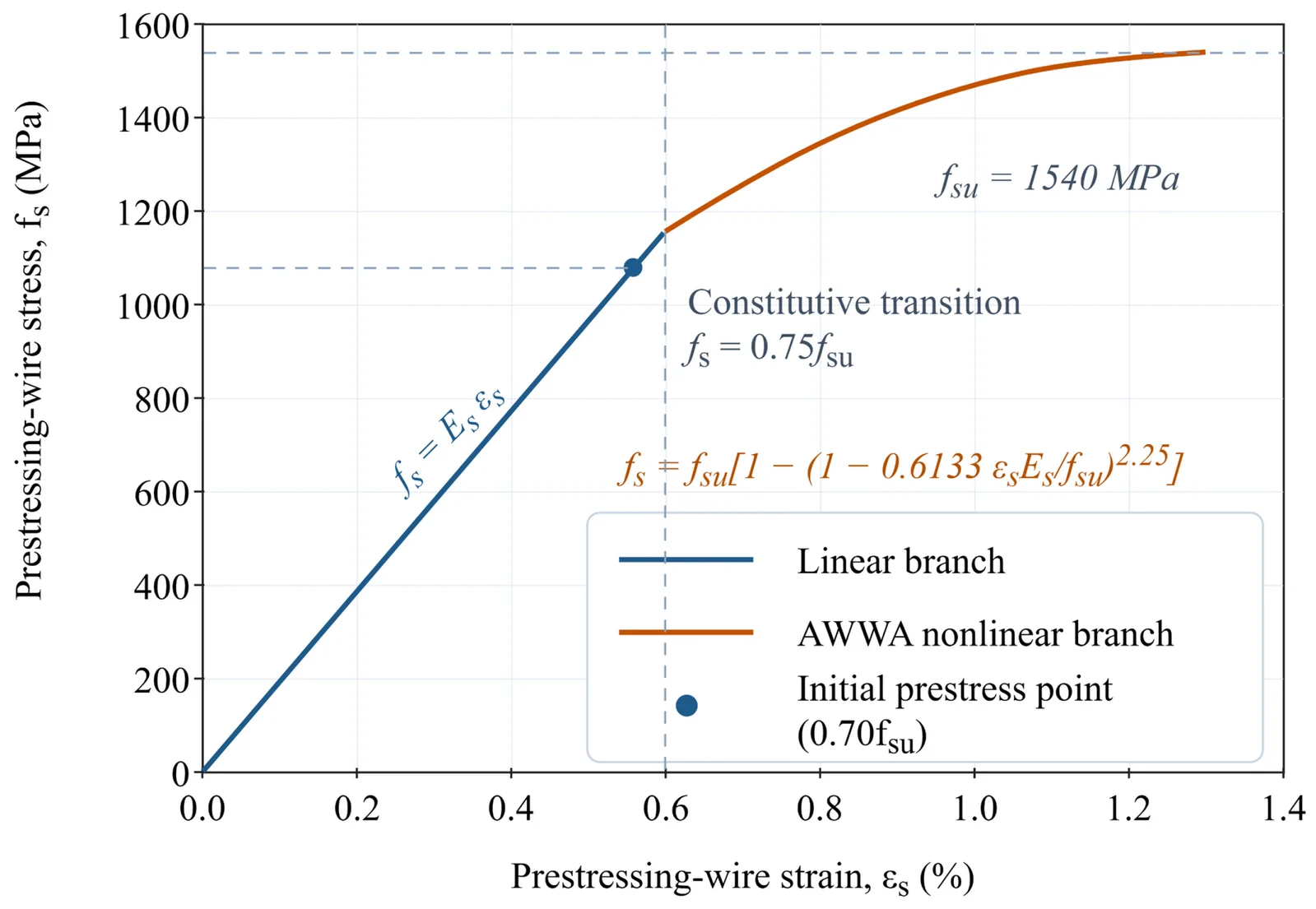
Piecewise stress–strain relationship of the prestressing wire.

The original steel cylinder and strengthening steel cylinder were represented using a two-stage ideal elastic–plastic model. The prestressing wires were modeled using the piecewise constitutive relation recommended by AWWA C304 [2], with $E_{s}=193,050$ MPa and a specified tensile strength of $f_{su}=1540$ MPa. The initial wire stress in the numerical model was set to $f_{sg}=0.7f_{su}=1078$ MPa, as shown in Figure 5.

### 3.2. Finite Element Model Establishment

Based on the geometric dimensions of the full-scale test pipe, three-dimensional geometric models of the concrete core, original steel cylinder, prestressing wires, mortar coating, strengthening steel cylinder, and micro-expansive concrete grouting layer were established in ABAQUS. The assembly followed the relative positions of the components in the test pipe. The pipe-core concrete, mortar coating, and grouting layer were discretized using eight-node three-dimensional linear reduced-integration solid elements (C3D8R). The original and strengthening steel cylinders were modeled using four-node linear reduced-integration shell elements (S4R). For the strengthening steel cylinder, the shell reference surface was located at its outer surface at a radius of 760 mm, and the shell thickness of 16 mm was offset inward in the radial direction, giving a physical inner radius of 744 mm. The prestressing wires were simulated using two-node three-dimensional linear truss elements (T3D2) [12]. The complete medium-mesh numerical model contained 50,438 nodes and 38,310 elements, including 17,828 solid elements, 6460 shell elements, and 14,022 truss elements. Boundary conditions were set according to the test support arrangement. Radial and circumferential displacement constraints were applied at the support regions to restrict rigid-body motion and maintain consistency with the test loading state.

The prescribed initial prestress was established in the wire elements before internal-pressure loading. The prestressed pipe was first brought into equilibrium, after which the broken-wire and pressure-loading steps were applied.

Wire breakage was prescribed through element removal. After the prestress-equilibration step, the selected wire elements were removed in the specified analysis step, releasing their stored tensile force. Removal was defined by the prescribed damage window and was not triggered by a wire-failure criterion.

### 3.3. Interface Connections and Contact Simplification

For interface connections in the original pipe, embedded or tie constraints were used between the original steel cylinder and the pipe-core concrete and between the prestressing wires and adjacent concrete/mortar layers. These constraints provide an equivalent description of displacement compatibility and global composite action among the original pipe layers at the scale of this study [14,15]. This treatment is used to simulate the overall working state of the multilayer original pipe, whereas local interlayer debonding and wire–mortar microslip are not the primary research objects.

Tie constraints were assigned to the strengthening-cylinder–grout and grout–original-core interfaces to represent dense grouting and bonded interfaces. The constraints enforce displacement compatibility between the connected surfaces. During post-repair loading to 1.6 MPa, no new through cracks or macroscopic instability was observed, and the measured component strains generally increased with water pressure. The calculated component load sharing and strain reductions correspond to the adopted tied-interface condition; interface opening, slip, and debonding were not included.

### 3.4. Model Validation

**Figure 6:**
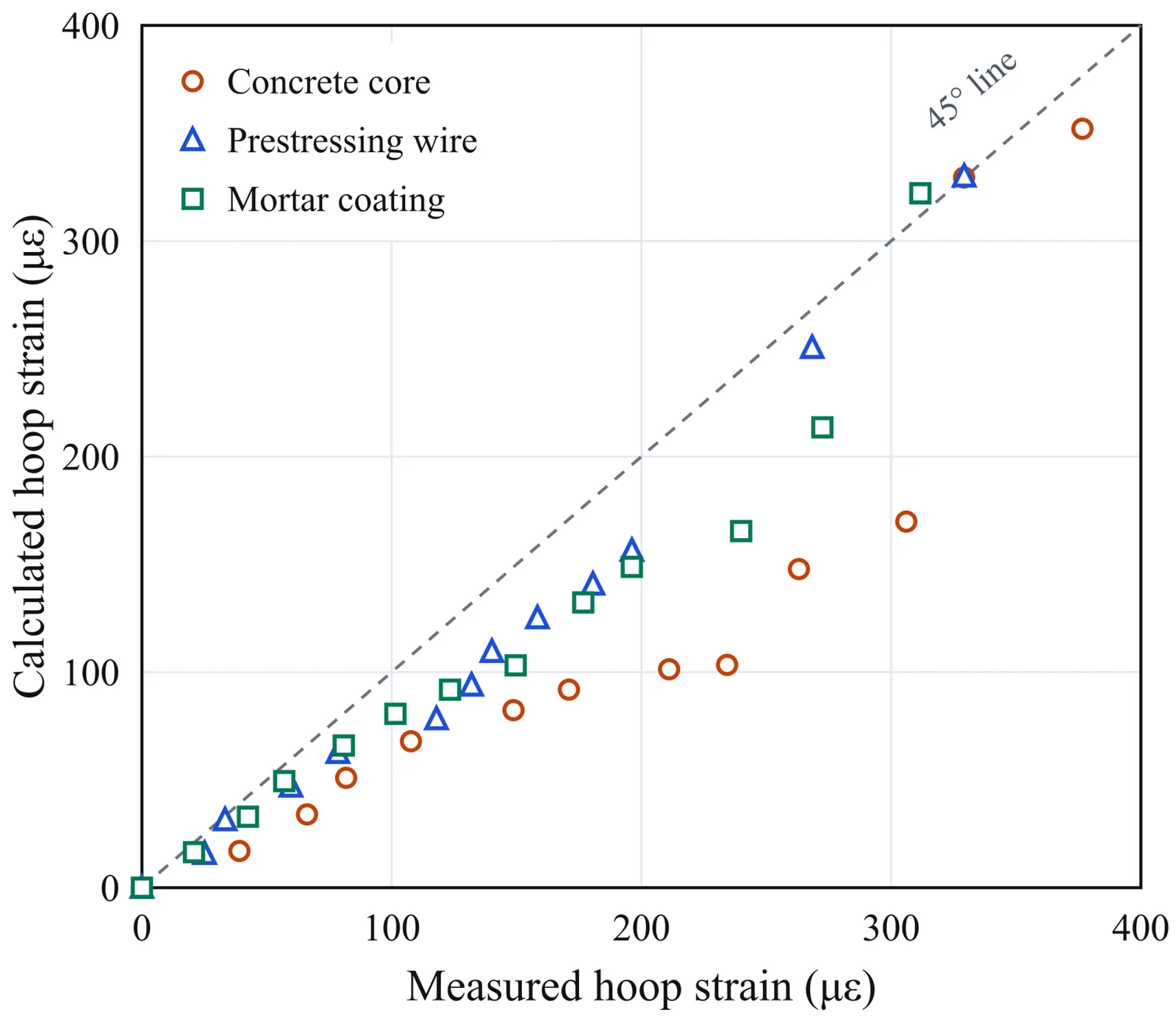
Comparison between measured and calculated hoop strains at the S1 measurement location for the concrete core, prestressing wire, and mortar coating. Each point represents a matched pressure level, and the dashed line denotes equality between the measured and calculated values.

For the original pipe before artificial wire cutting, numerical strains were extracted at the S1 measurement location using the same axial position, circumferential position, structural component, and hoop-strain direction as the corresponding experimental gauges. Figure 6 compares the measured and calculated strains of the concrete core, prestressing wire, and mortar coating at 13 matched pressure levels from 0 to 1.2 MPa. Each point represents the experimental and numerical results at the same pressure level, and the 45° reference line denotes equality between the two values.

For the concrete core, the calculated and measured strains are close during the initial loading stage and at the final pressure level, while several intermediate-pressure points deviate from the 45° reference line. This intermediate stage corresponds to the progressive development of local cracks. The measured point strain is influenced by aggregate distribution, local material heterogeneity, and the position of nearby cracks relative to the strain gauge, whereas the homogenized finite-element representation produces a smoother regional response.

The prestressing-wire and mortar-coating results are generally distributed near the 45° reference line. Their calculated strains increase consistently with the measured values and reproduce the strain development throughout the loading process. The point-by-point comparison provides the calibration basis for the baseline numerical response.

### 3.5. Key Simulation Conditions

Based on the finite-element model established above, parametric simulations were conducted with nominal wire breakage level and damage location as the principal variables. The nominal wire breakage levels were 5%, 15%, and 25%, and the two representative locations were the pipe midspan and pipe-end socket region.

The 5%, 15%, and 25% values are nominal numerical levels used to compare the influence of increasing local prestress loss. The repaired approximately 5% configuration corresponds to the post-repair hydrostatic-pressure test, whereas the unrepaired 5% configuration and all 15% and 25% configurations are numerical parametric extensions evaluated using the test-calibrated model.

The 5%, 15%, and 25% values represent nominal numerical damage levels implemented using contiguous local wire-removal windows. The pipe-midspan windows were centered on the midspan region, whereas the pipe-end socket windows extended inward from the pipe end. The axial extent of the contiguous window increased with the nominal damage level, and the same spatial construction rule was used for all compared configurations. The pipe-end socket region is affected by the end-ring configuration, interruption of prestressing-wire wrapping, and boundary constraints; comparison with the pipe midspan therefore identifies the influence of location on the calculated response.

**Figure 7:**
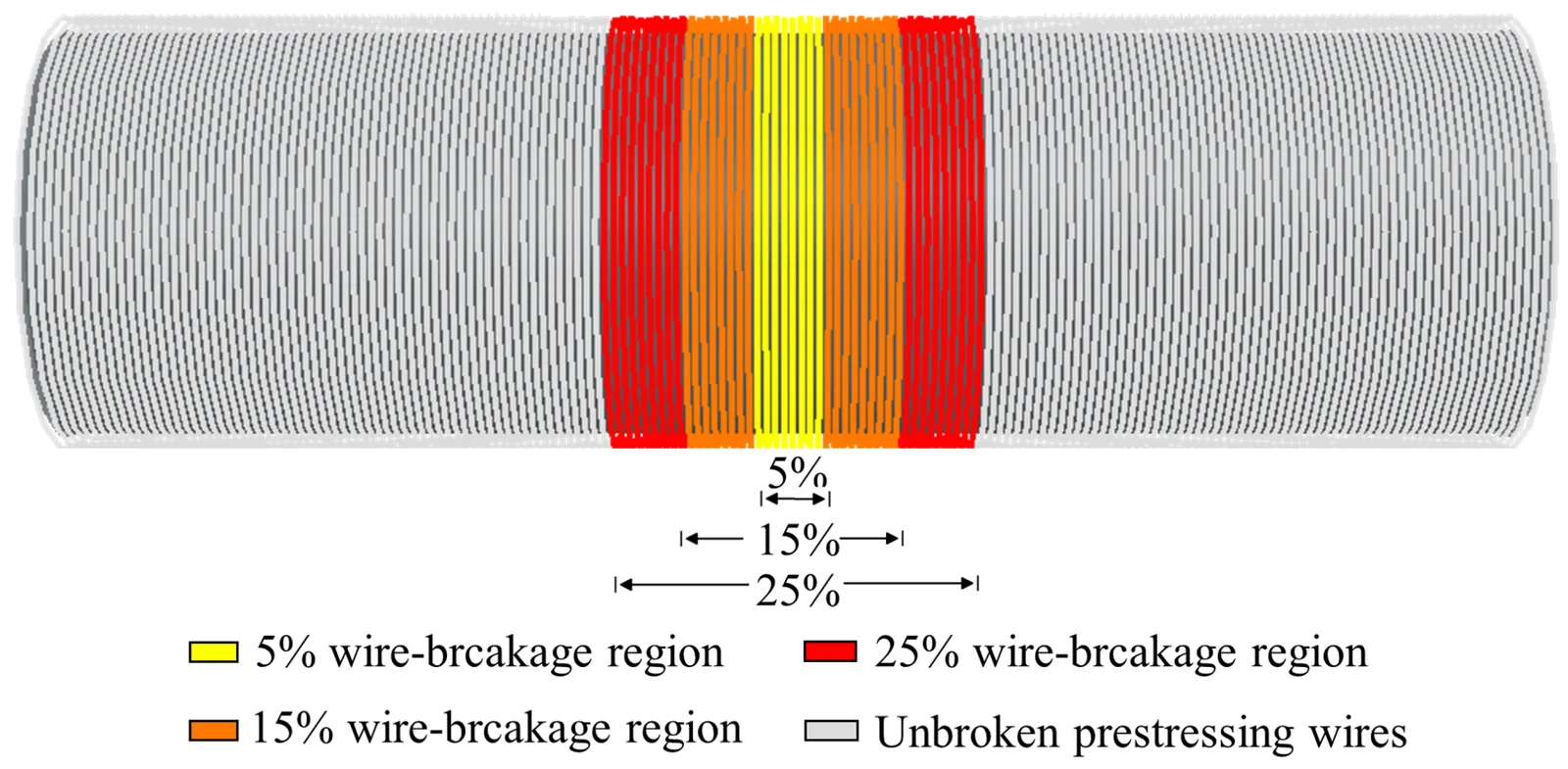
Setup of damage windows at the pipe midspan under different nominal wire breakage levels.

**Figure 8:**
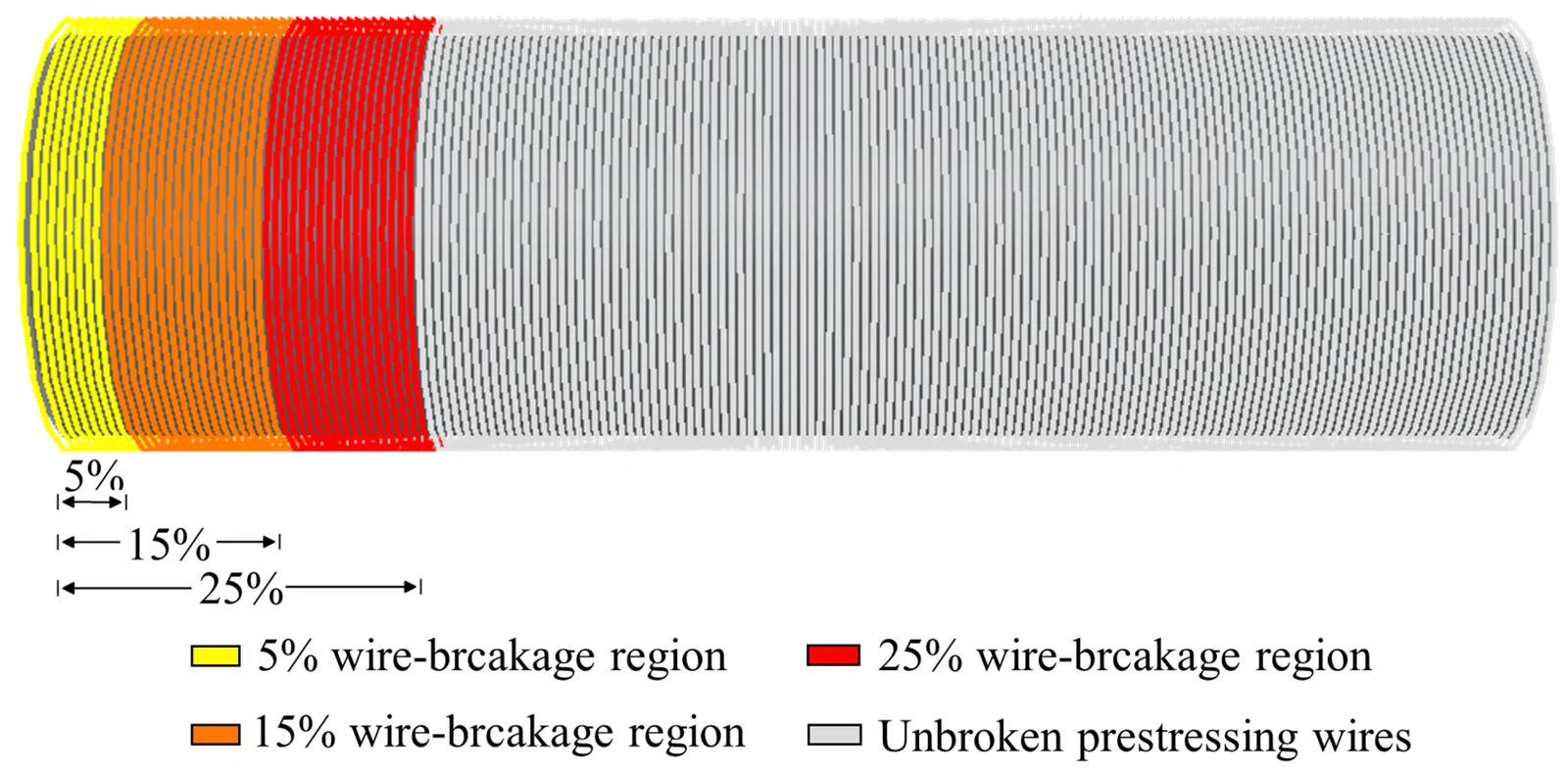
Setup of damage windows at the pipe-end socket region under different nominal wire breakage levels.

The prescribed damage-window settings for the pipe midspan and pipe-end socket region are shown in Figure 7 and Figure 8, respectively.

In the response-curve identifiers, *L* and *M* denote the pipe-end socket region and pipe midspan, respectively; 12 and 16 denote maximum analysis pressures of 1.2 and 1.6 MPa; and 5, 15, and 25 denote the nominal broken-wire levels. The suffixes s1 and s2 denote the original steel cylinder and strengthening steel cylinder, respectively, while an identifier without a suffix refers to the concrete core.

### 3.6. Mesh-Sensitivity Analysis

The unrepaired 25% pipe-midspan configuration was selected for mesh assessment because it contains a severe prescribed damage window and a pronounced nonlinear response. The coarse (C), medium (M), and fine (F) models retained the same geometry, nominal damage level, materials, boundary conditions, loading steps, and output regions. Their mesh sizes and results at 1.2 MPa are summarized in Table 3.

From the medium to the fine mesh, the concrete-core regional mean strain increment changes by 0.12%, the original-cylinder regional mean strain increment by 2.43%, and the concrete-core damaged-volume ratio by 0.54%. The maximum value of (ALLAE+ALLSD)/ALLIE is 0.128%, below 0.13%. These regional measures support the medium mesh for comparative regional-response analysis within the investigated configuration. Localized post-cracking maxima are treated as mesh-dependent calculated responses and are not used as stand-alone measures of material limits or global load-transfer mechanisms.

## 4. Results and Discussion

### 4.1. Nonlinear Damage Evolution of the Concrete Core and Crack-Suppression Mechanism

The concrete core is the main load-bearing and stiffness component of PCCP. Its cracking and damage propagation directly affect structural stiffness degradation and subsequent load-transfer paths. This section examines the concrete-core hoop-strain response before and after repair under different broken-wire conditions and evaluates the effects of damage level and location using strain-response curves, sensitivity analysis, and spatial damage distributions.

**Figure 9:**
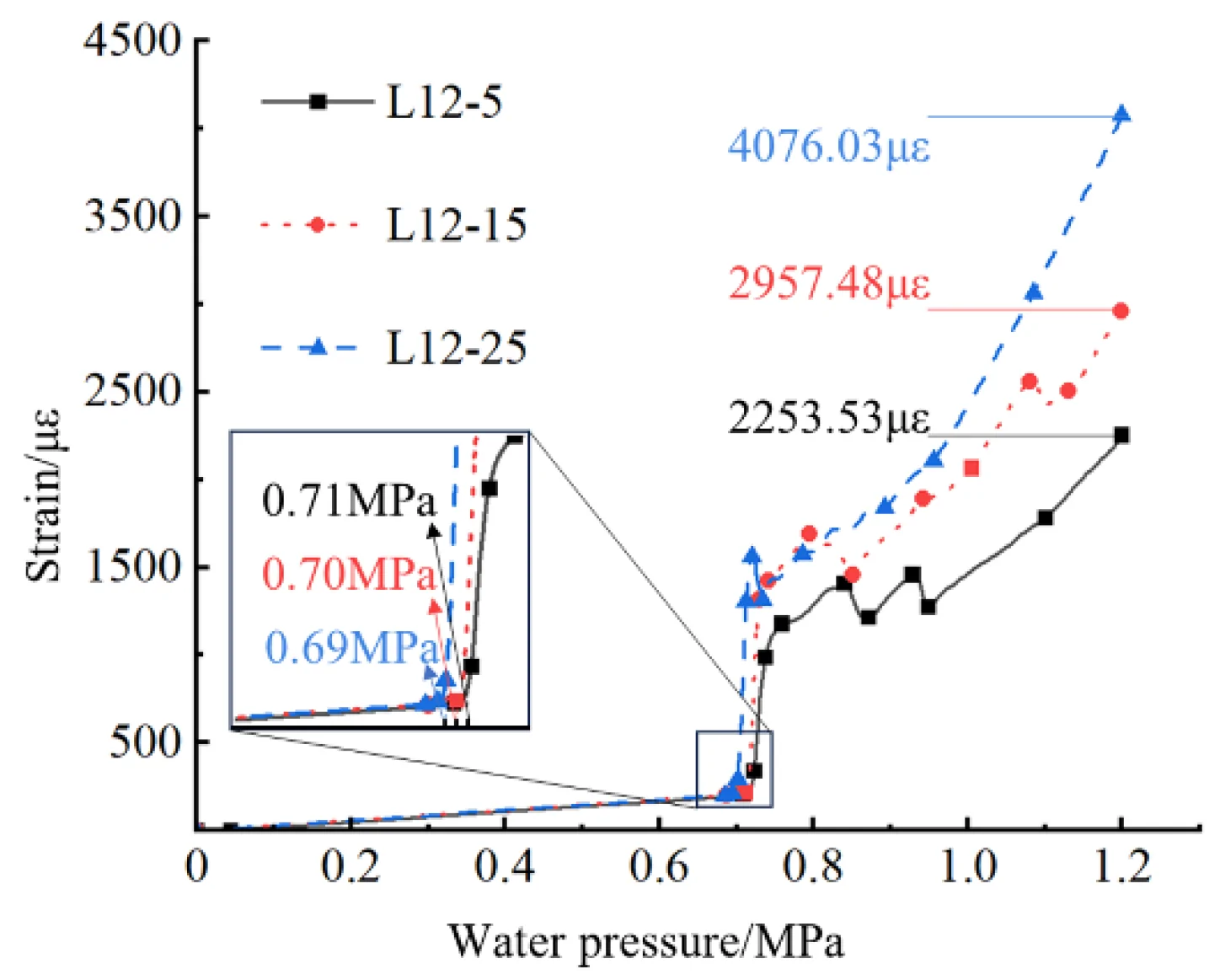
Strain–water pressure curves of the concrete core at the pipe-end socket region under different wire breakage ratios.

**Figure 10:**
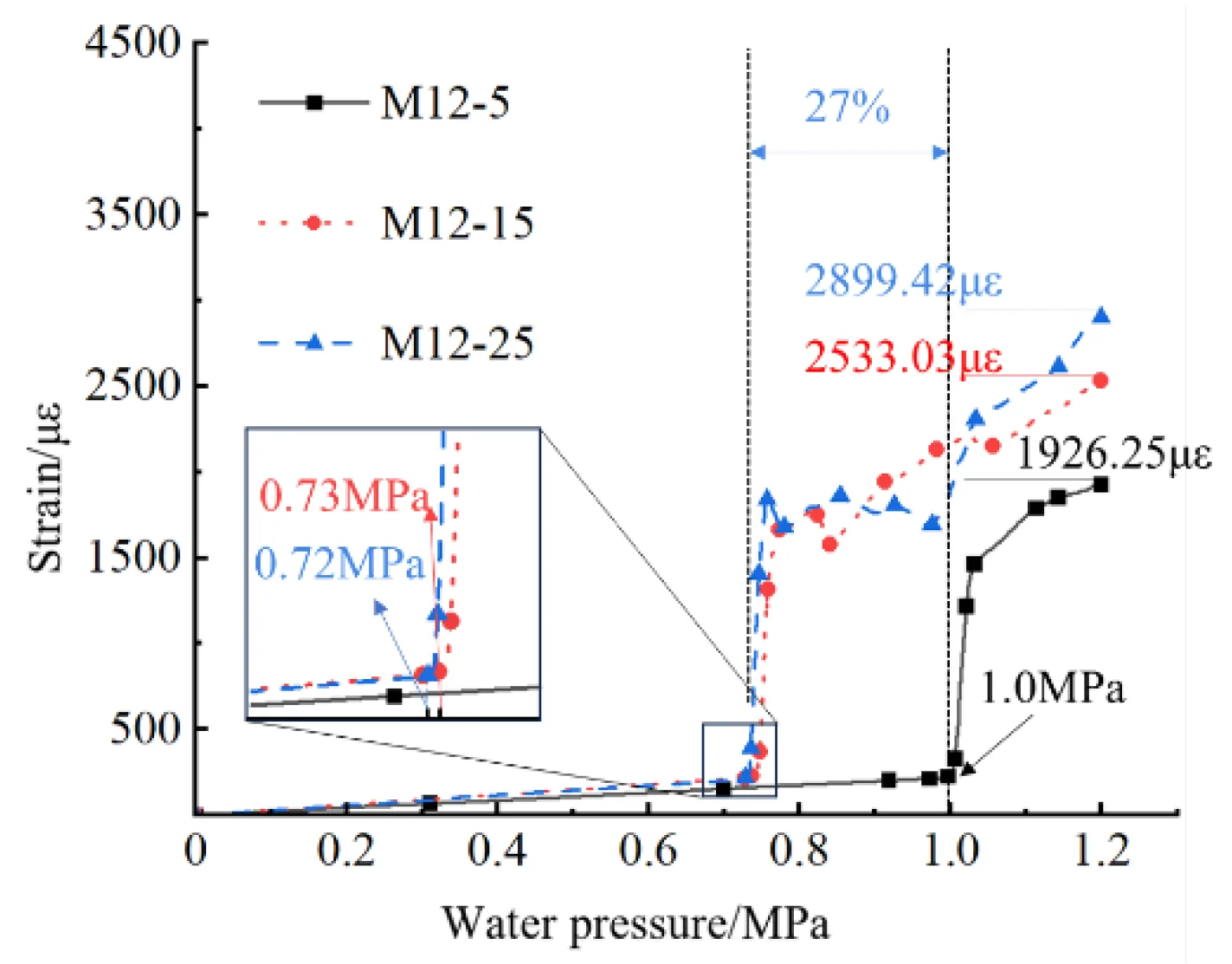
Strain–water pressure curves of the concrete core at the pipe midspan under different wire breakage ratios.

Figure 9 and Figure 10 show that all broken-wire conditions exhibit approximately linear growth in the early loading stage, indicating that the prestressing wires, concrete core, and original steel cylinder still work together at this stage. For the numerical configurations, the pressure range associated with a pronounced change in slope of the concrete-core strain curve was used to characterize the transition from the initial approximately linear stage to the nonlinear-response stage. As internal water pressure continues to increase, the slopes of the strain curves increase markedly in some conditions, reflecting the nonlinear response of the concrete core under these conditions.

At the pipe-end socket region, the concrete-core strain curves for the 5%, 15%, and 25% broken-wire configurations enter the nonlinear-response stage at approximately 0.7 MPa. The similar transition pressures indicate that the pipe-end socket response is sensitive to the prescribed loss of circumferential restraint. At 1.2 MPa, the calculated concrete-core strains at the pipe-end socket region are 2253.53, 2957.48, and 4076.03 με, respectively.

At the pipe midspan, the 5% broken-wire configuration enters the nonlinear-response stage near 1.0 MPa. For the 15% and 25% configurations, the corresponding numerical transition occurs near 0.7 MPa as the prescribed loss of circumferential restraint increases. The calculated concrete-core strains at 1.2 MPa are 1926.25, 2533.03, and 2899.42 με for the 5%, 15%, and 25% configurations, respectively. These values are approximately 14.5%, 14.3%, and 28.9% lower than those at the pipe-end socket region.

These differences indicate that the damage response of the pipe-end socket region is affected not only by wire breakage ratio but also by the end configuration, interruption of prestressing-wire wrapping, and boundary constraints. The pipe midspan is mainly controlled by the weakening of local circumferential precompression caused by increasing wire breakage ratio, which further amplifies the local strain response.

**Figure 11:**
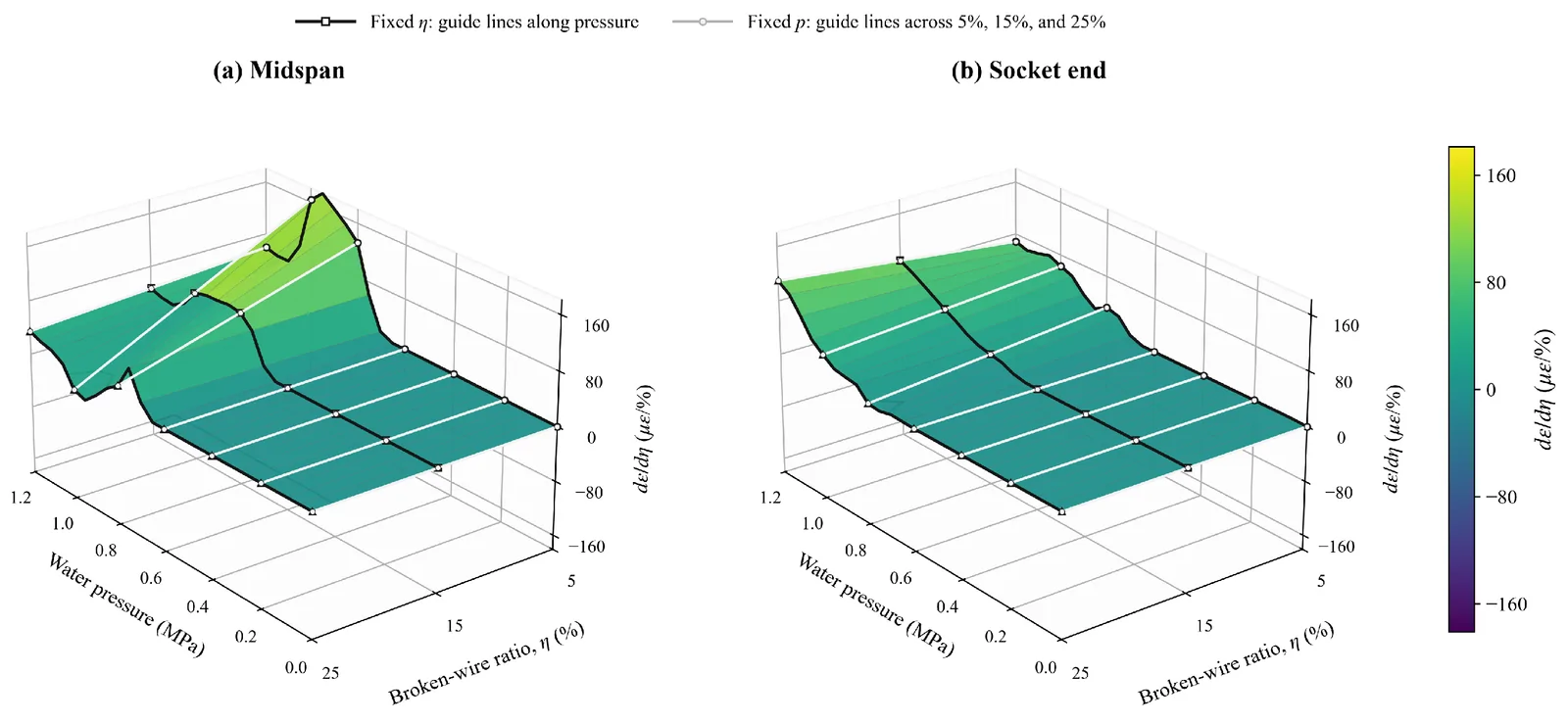
Strain-sensitivity surface of hoop strain in the unrepaired concrete core with coordinate-aligned guide lines: (**a**) pipe midspan; (**b**) pipe-end socket region.

To characterize the coupled effects of wire breakage ratio, water pressure, and damage location on concrete-core response, a hoop-strain sensitivity surface was constructed from the unrepaired results, as shown in Figure 11. Because only three discrete wire breakage ratios were considered, the sensitivity index is not a continuous derivative. It is an interval average calculated by finite difference, $\Delta \varepsilon_{c}/\Delta \eta$, where $\varepsilon_{c}$ is the hoop strain of the concrete core in με and η is the wire breakage ratio in percent. Its unit is με/%, representing the average strain change for each one-percentage-point increase in wire breakage ratio rather than a percentage growth rate. To improve quantitative readability, coordinate-aligned guide lines were superimposed on the sensitivity surface. The dark longitudinal lines trace the sensitivity responses at nominal broken-wire ratios of 5%, 15%, and 25% over the pressure range. The light transverse lines connect the three broken-wire-ratio positions at water pressures of 0.0, 0.2, 0.4, 0.6, 0.8, 1.0, and 1.2 MPa. Markers identify the intersections of the two sets of guide lines.

Figure 11 shows that concrete-core strain sensitivity is generally low in the low-pressure stage. As water pressure increases and the core enters the cracking and stiffness-degradation stage, the strain differences among wire breakage ratios are amplified. A local high-sensitivity region appears at the pipe midspan at approximately 0.90–1.00 MPa. By comparison, the high-sensitivity region of the pipe-end socket region shifts toward the high-pressure stage and remains high near 1.20 MPa.

Negative values indicate that, at the corresponding pressure level and broken-wire-ratio interval, the concrete-core strain does not increase monotonically with nominal broken-wire ratio; they do not represent structural recovery.

**Figure 12:**
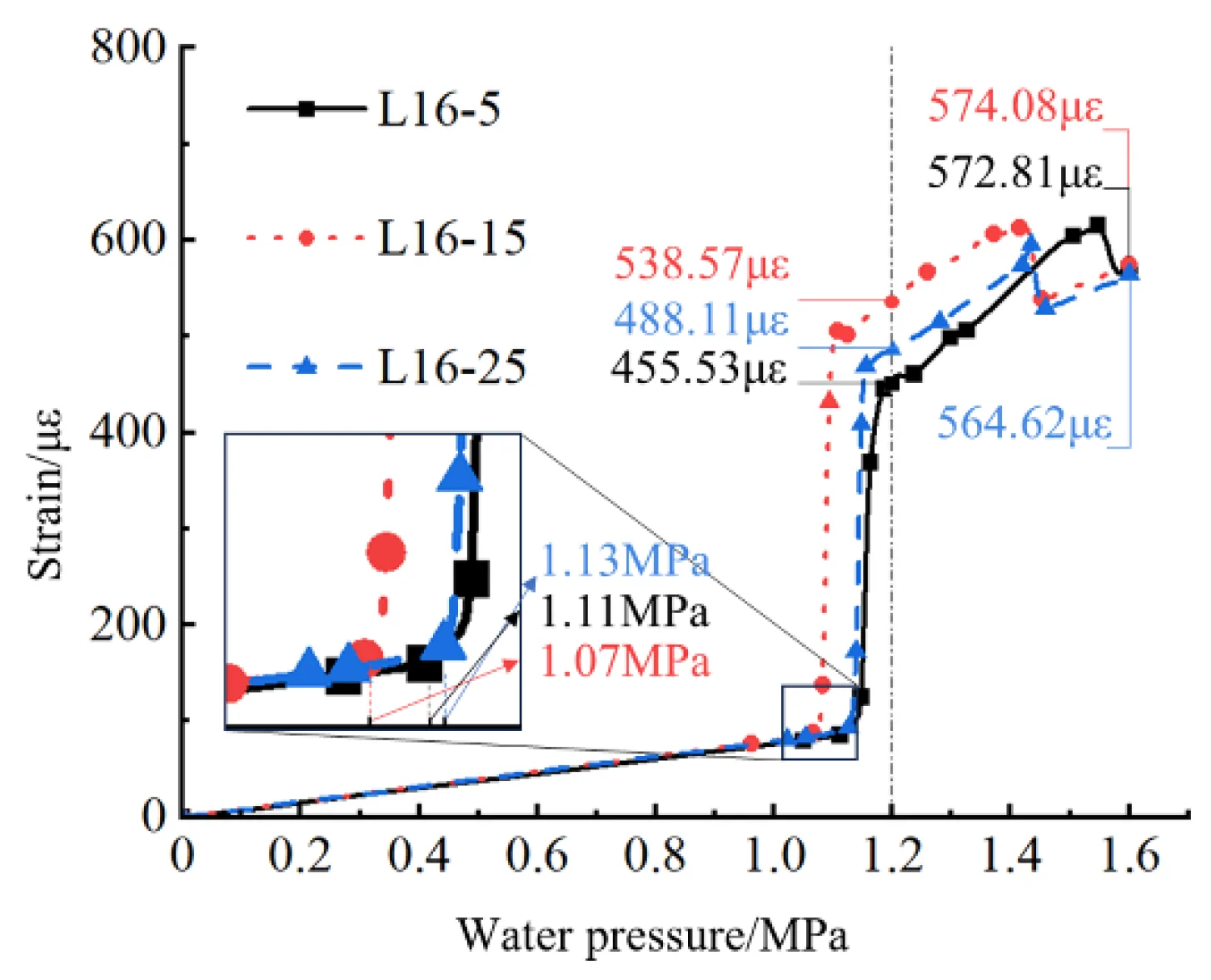
Strain response curves of the concrete core at the pipe-end socket region after strengthening.

**Figure 13:**
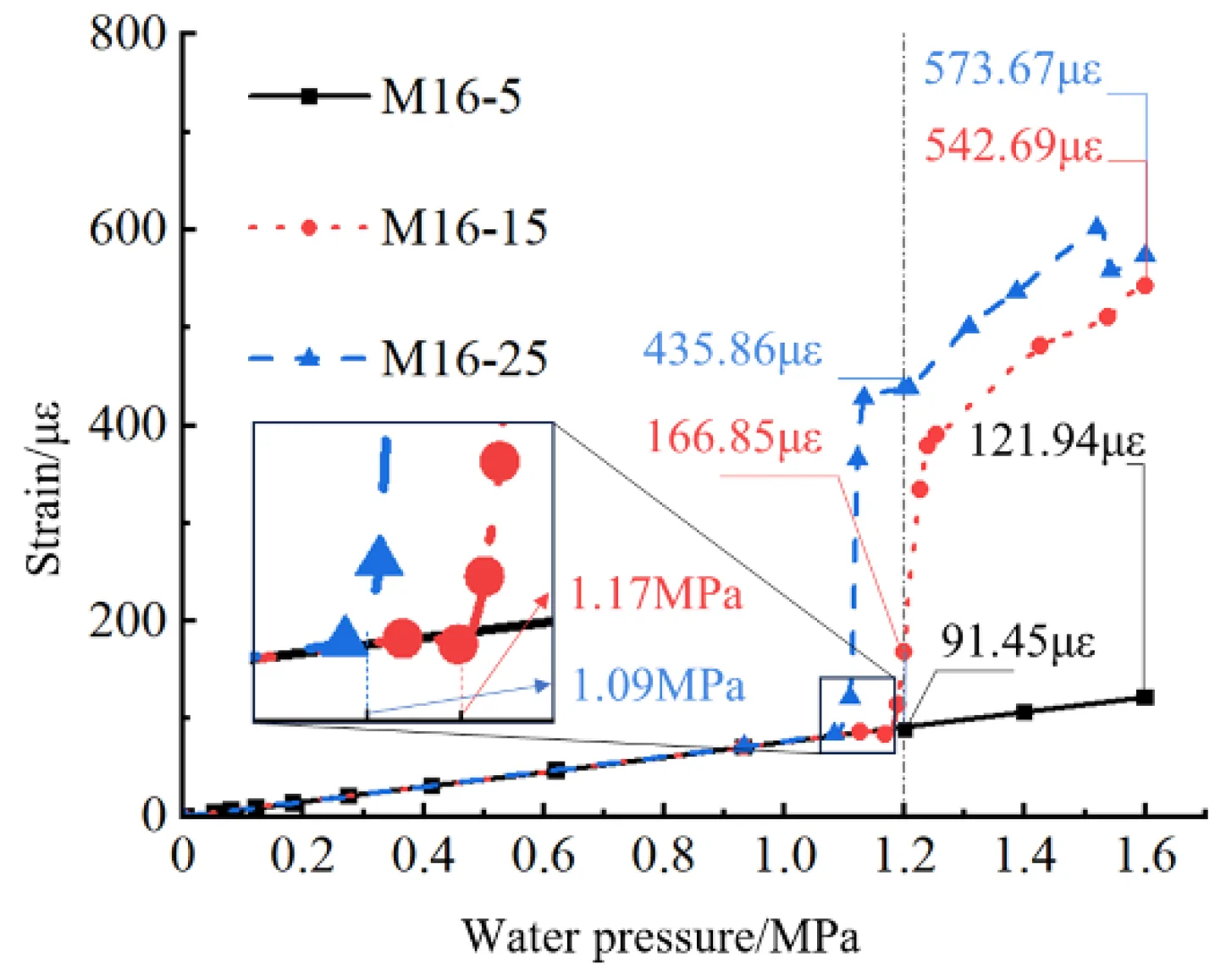
Strain response curves of the concrete core at the pipe midspan after strengthening.

After the strengthening steel cylinder–micro-expansive concrete grouting composite repair system is introduced, the strain response of the concrete core decreases markedly and the curve slopes generally become gentler. Figure 12 and Figure 13 show that after repair, all conditions maintain slow strain growth below 1.0 MPa.

Repair responses still differ by damage location. For the pipe-end socket conditions, all three wire breakage ratio curves show a sudden strain increase near 1.1 MPa, reflecting a localized nonlinear response influenced by the end configuration and boundary constraints. However, at the working water pressure of 1.2 MPa, the concrete-core strains remain relatively low, with values of 455.53, 538.57, and 488.11 με for the 5%, 15%, and 25% conditions, respectively. When loading continues to 1.6 MPa, the final pipe-end socket core strains under different wire breakage ratios fall within the range of 564.62–574.08 με, showing that the repair system weakens the influence of wire breakage ratio on pipe-end socket response.

At the pipe midspan, the concrete-core strains under the 5% and 15% broken-wire configurations are approximately 91.45 and 166.85 με at 1.2 MPa, respectively. For the 25% configuration, the numerical curve enters the nonlinear-response stage near 1.1 MPa, and the strain at 1.2 MPa is 435.86 με.

**Figure 14:**
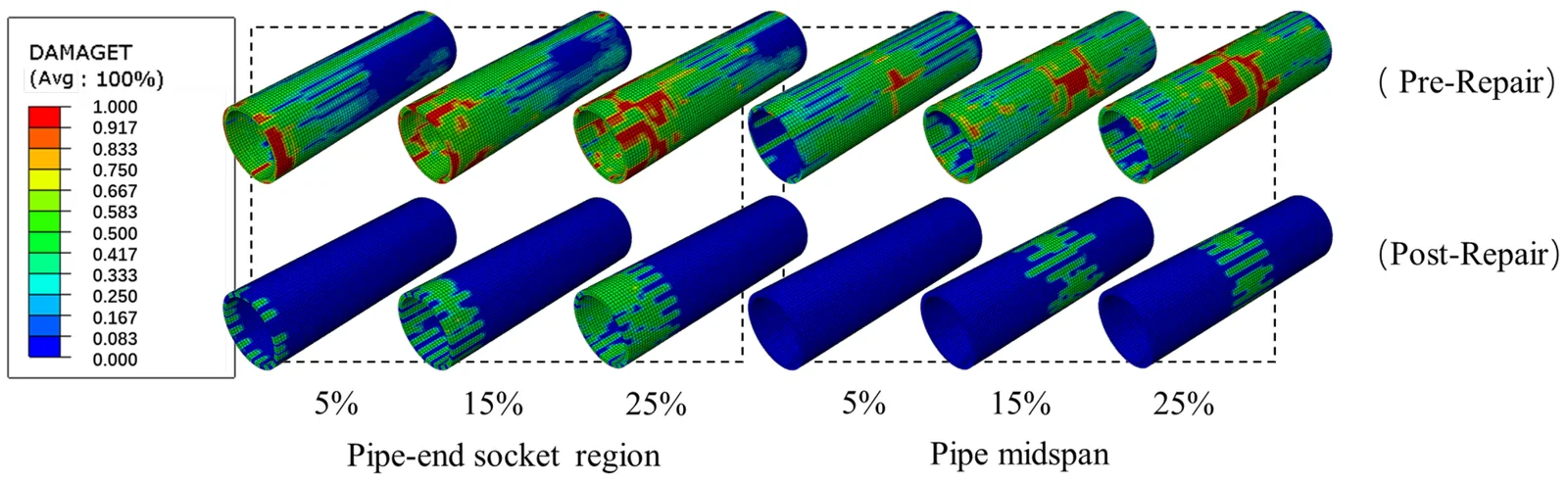
Comparison of tensile damage factor (DAMAGET) of the concrete core before and after strengthening at a design water pressure of 1.2 MPa under different wire breakage conditions: (**top**) before strengthening; (**bottom**) after strengthening.

Figure 14 further confirms these patterns from the spatial damage distribution. Before repair, high values of the tensile damage factor in the concrete core are mainly concentrated in and near the damage window, and the pipe-end socket condition is more prone to local damage concentration. After repair, the high-damage region contracts markedly, and the overall core remains mainly in a low-damage state except near the damaged-wire area.

Previous studies have shown that the number and location of broken wires affect the structural response of PCCP [8,9]. Numerical analysis of clustered wire loss and full-scale internal-pressure testing have also reported progressive changes in stress and deformation as wire damage develops [13,14]. The expansion of the affected concrete-core region and the increase in original steel-cylinder demand obtained in the present study are consistent with these trends. The present comparison further shows that, under the same nominal damage level, the pipe-end socket region develops a stronger local response than the pipe midspan, reflecting the geometric and boundary differences between the two locations.

### 4.2. Unloading Effect of the Original Steel Cylinder and Load-Transfer Redistribution

With local failure of prestressing wires and development of core cracking, circumferential load is redistributed among the prestressing wires, concrete core, and original steel cylinder, resulting in a significant increase in the hoop tensile strain of the original steel cylinder. The strain evolution and stress distribution of the original steel cylinder therefore reflect the redistribution of circumferential load in the original pipe after broken-wire damage and are important indicators for evaluating the unloading effect of internal steel-cylinder repair. This section extracts hoop-strain curves and stress contours of the original steel cylinder before and after repair to analyze the influences of wire breakage ratio, damage location, and repair measures on its stress state.

**Figure 15:**
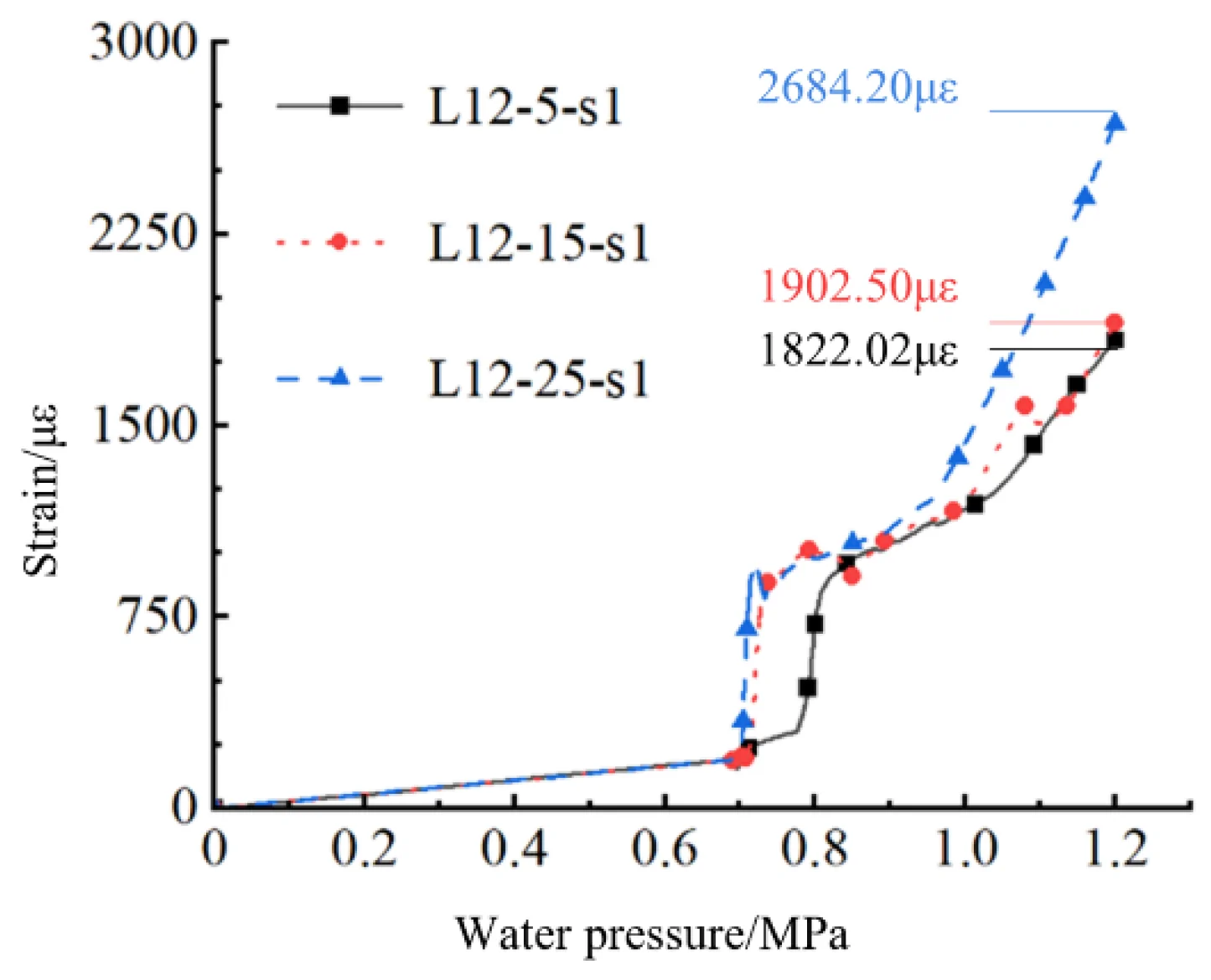
Strain–water pressure curves of the original steel cylinder at the pipe-end socket region before strengthening.

**Figure 16:**
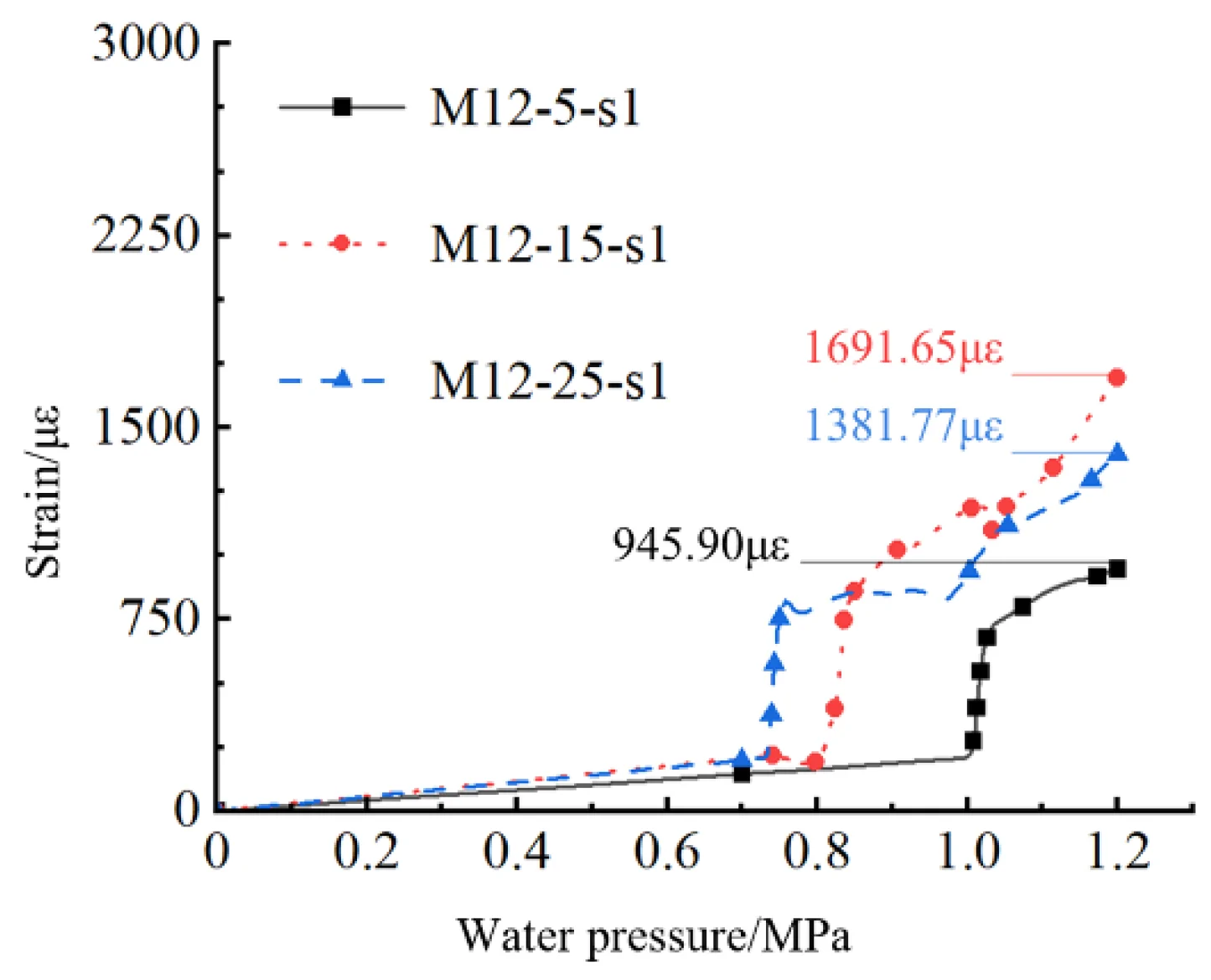
Strain–water pressure curves of the original steel cylinder at the pipe midspan before strengthening.

Figure 15 and Figure 16 show that, in the unrepaired state, the original steel-cylinder strain exhibits clear staged behavior with increasing water pressure. In the early loading stage, the concrete core and prestressing wires still provide strong circumferential restraint, and the original steel-cylinder strain increases slowly. After the concrete core enters the cracking-damage stage, a larger share of the circumferential load is transferred to the original steel cylinder, causing its strain to increase rapidly. Using the nominal yield strain of Q235 steel, approximately 1141 με, as the reference for the original steel cylinder, the pipe-midspan 5% broken-wire condition remains within the elastic range at 1.2 MPa, with a strain of 945.90 με. When the wire breakage ratio increases to 15% and 25%, the strains reach 1691.65 and 1381.77 με, respectively. At the pipe-end socket region, the strains reach 1822.02, 1902.50, and 2684.20 με under the 5%, 15%, and 25% conditions, respectively.

At the pipe-end socket region, the fixed-position strain levels for the 5% and 15% configurations are close, whereas the strain under the 25% configuration increases markedly. At the pipe midspan, the fixed-position original-cylinder strain in the 15% case is higher than that in the 25% case. This comparison describes the response at the selected output position and is reported as a local non-monotonic result. It is not used to infer a general transition in structural load transfer.

**Figure 17:**
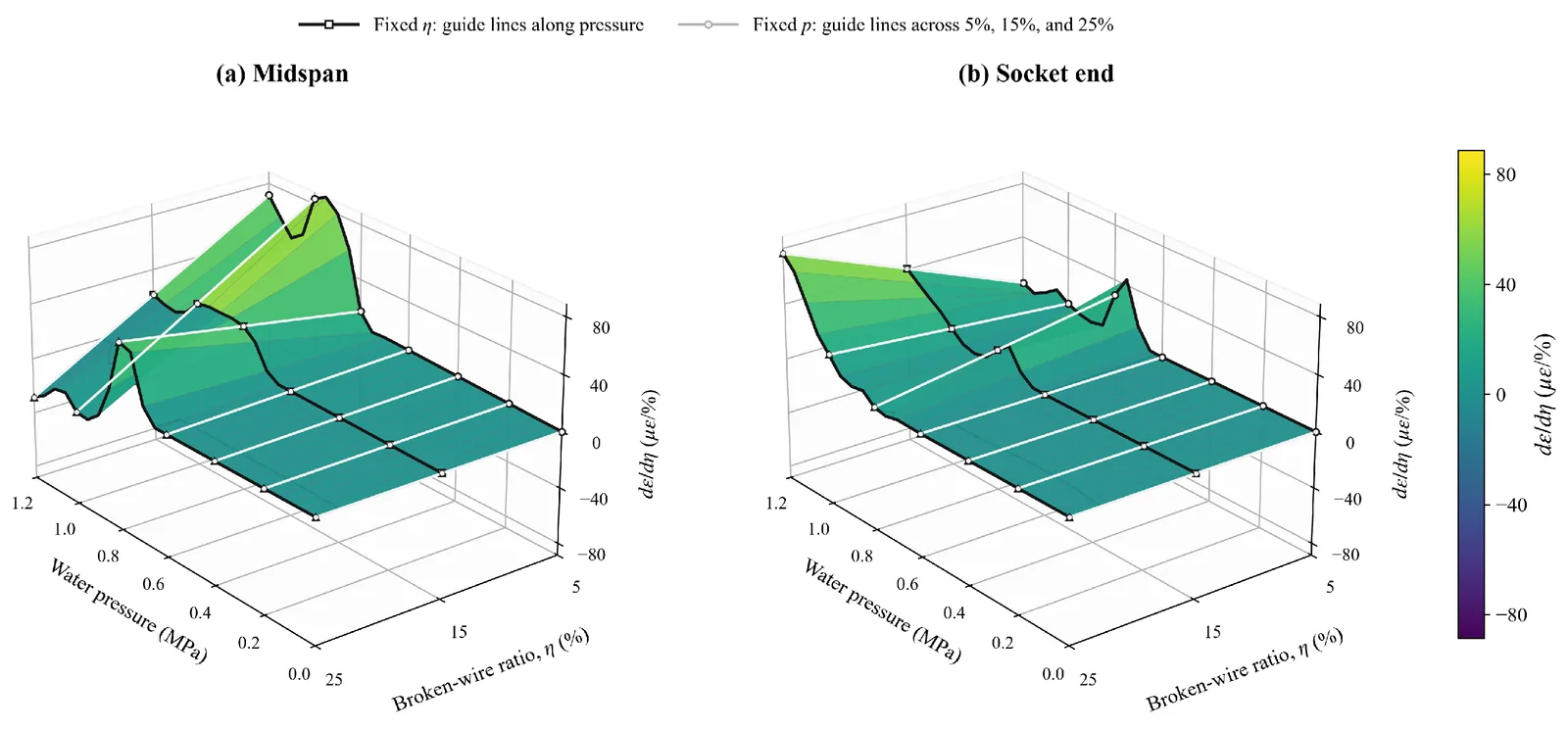
Strain-sensitivity surface of hoop strain in the unrepaired original steel cylinder with coordinate-aligned guide lines: (**a**) pipe midspan; (**b**) pipe-end socket region.

To further reveal the influence of wire breakage ratio on the original steel-cylinder response, a hoop-strain sensitivity surface was constructed from the unrepaired strain results, as shown in Figure 17. Using the same finite-difference definition as Figure 11, the index is $\Delta \varepsilon_{s}/\Delta \eta$, where $\varepsilon_{s}$ is the hoop strain of the original steel cylinder in με and η is the wire breakage ratio in percent. The coordinate-aligned guide lines follow the same definition as those in Figure 11, enabling the pressure-dependent response at each nominal broken-wire ratio and the differences among the three ratio positions at fixed water-pressure levels to be followed directly.

Figure 17 shows that the original steel-cylinder hoop-strain sensitivity remains relatively low during the low-pressure stage and increases as the concrete core enters the nonlinear-response stage. A high-sensitivity region appears at the pipe midspan at approximately 0.90–1.00 MPa, where load transfer to the original steel cylinder increases as the concrete-core response becomes nonlinear. The pipe-end socket region shows stronger sensitivity under high water pressure and high wire breakage ratio because geometric discontinuity and boundary constraints amplify the local hoop strain.

Negative values indicate that, at the corresponding pressure level and broken-wire-ratio interval, the selected fixed-position original steel-cylinder strain decreases between adjacent nominal damage cases. This is a local non-monotonic response and does not represent structural recovery, a reduction in the full-field response, or a general change in the load-transfer mechanism.

**Figure 18:**
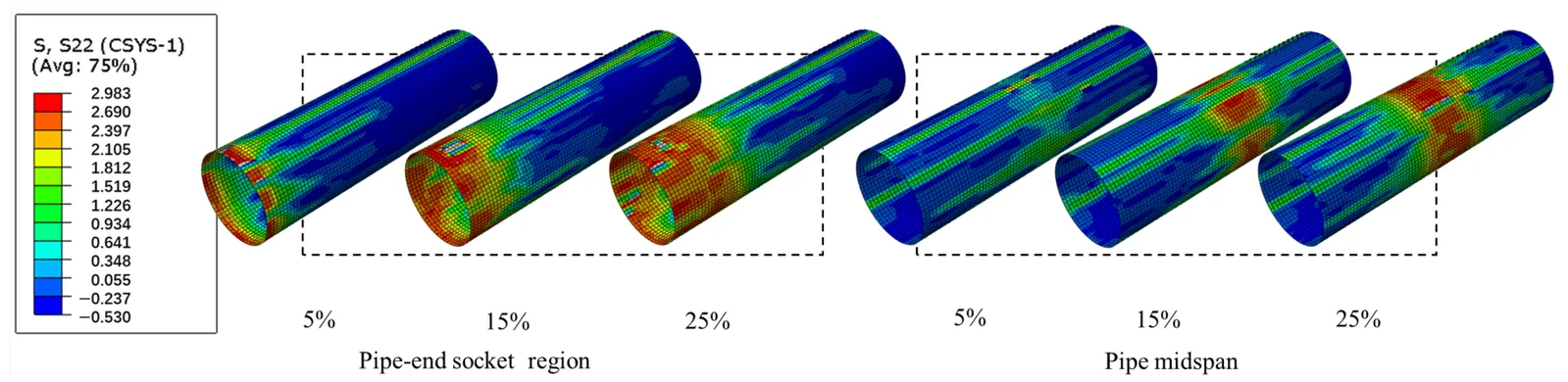
Hoop-stress distributions of the original steel cylinder in the unrepaired numerical configurations at 1.2 MPa. Each panel uses its labeled color scale to resolve the spatial stress gradient within the corresponding configuration.

Figure 18 shows the hoop-stress distributions of the original steel cylinder at a design water pressure of 1.2 MPa for different broken-wire conditions. Under the pipe-midspan 5% condition, the global stress level of the steel cylinder is consistent with the strain-curve result of 945.90 με. As the nominal wire breakage level increases, high-stress zones develop near the prescribed damage window. At the pipe-end socket region, the high-stress region is concentrated near the end and expands along the damaged area.

**Figure 19:**
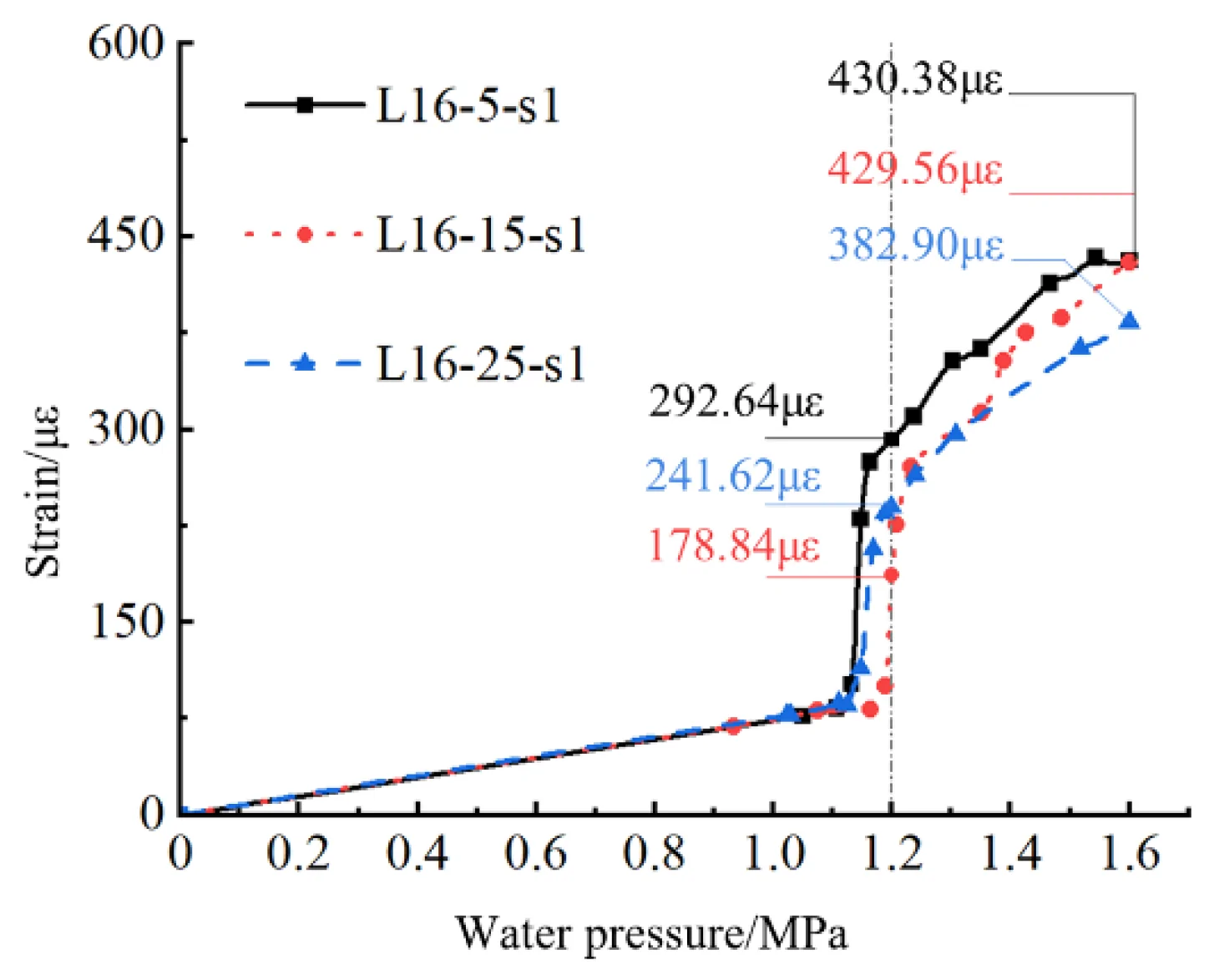
Strain–water pressure curves of the original steel cylinder at the pipe-end socket region after strengthening.

**Figure 20:**
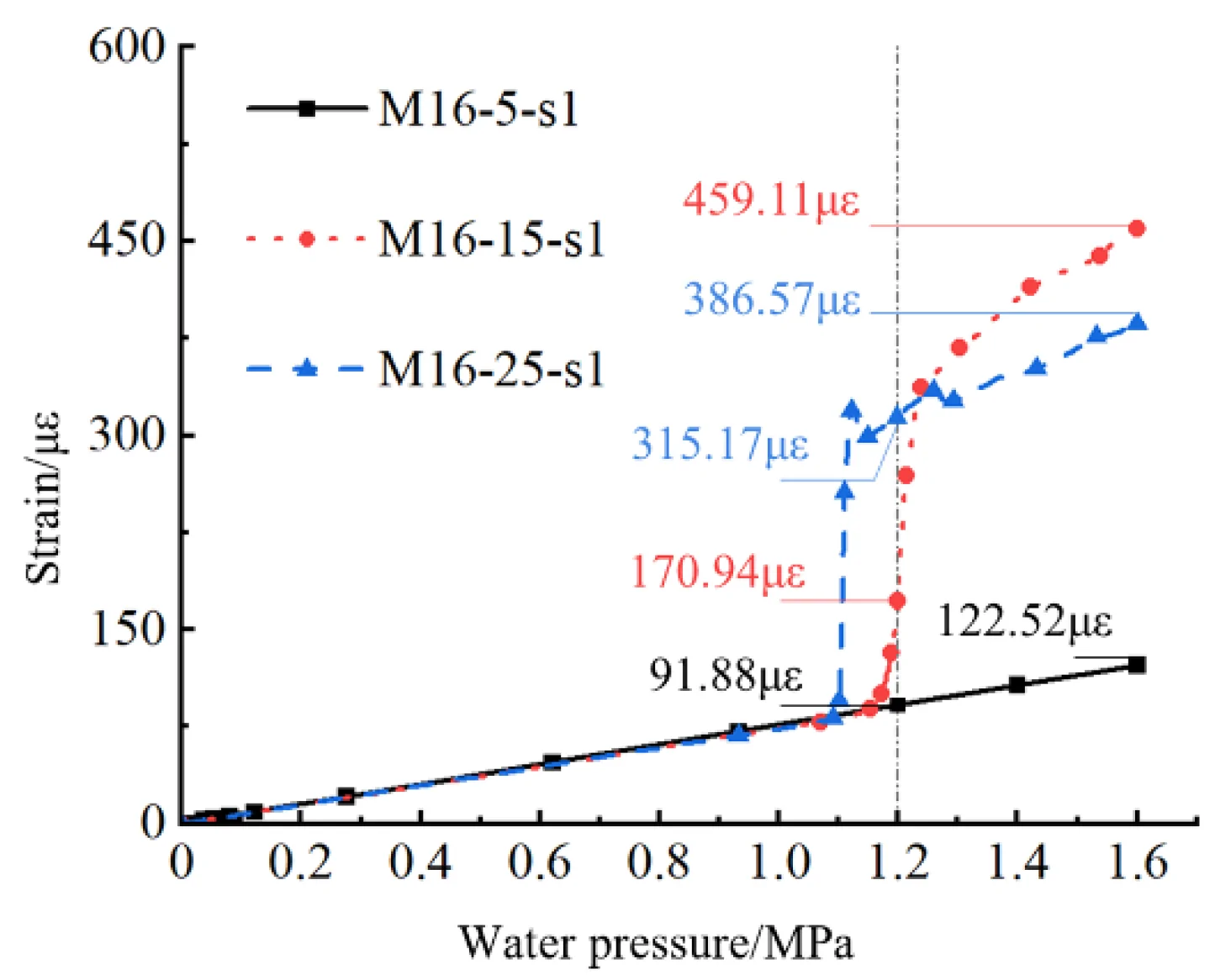
Strain–water pressure curves of the original steel cylinder at the pipe midspan after strengthening.

After the strengthening steel cylinder–micro-expansive concrete grouting composite repair system is adopted, the original steel-cylinder strain decreases significantly. Figure 19 and Figure 20 show that, at a water pressure of 1.2 MPa, the original steel-cylinder strains under the pipe-end socket 5%, 15%, and 25% conditions decrease to 292.64, 178.84, and 241.62 με, corresponding to reductions of 83.9%, 90.6%, and 91.0%, respectively. At the pipe midspan, the corresponding strains decrease to 91.88, 170.94, and 315.17 με, corresponding to reductions of 90.3%, 89.9%, and 77.2%, respectively. Even when the water pressure increases to 1.6 MPa, the maximum original steel-cylinder strain does not exceed 459.11 με and remains well below the reference yield strain, demonstrating that the repair system effectively reduces the hoop tensile strain developed in the original steel cylinder.

**Figure 21:**
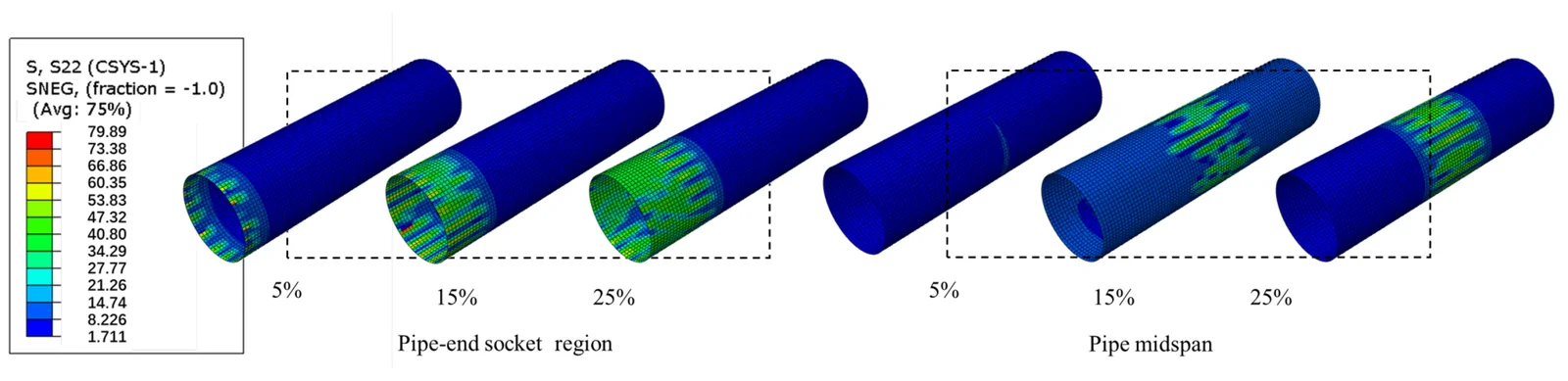
Hoop-stress distributions of the original steel cylinder in the repaired numerical configurations. Each panel uses its labeled color scale to resolve the spatial stress gradient within the corresponding configuration; quantitative comparisons with the unrepaired configurations are based on the response curves.

**Figure 22:**
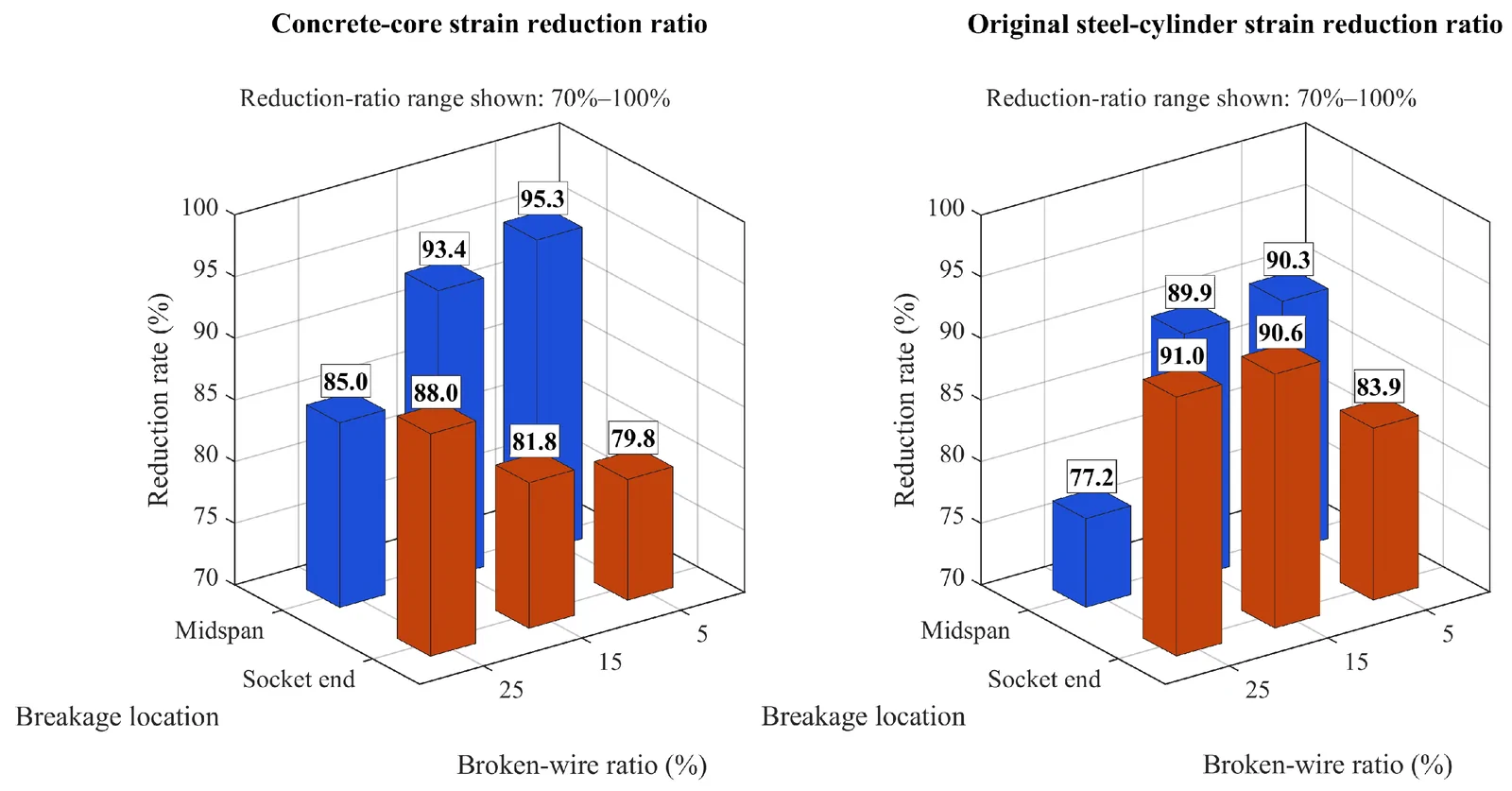
Reduction ratios of concrete-core strain and original steel-cylinder strain after internal steel-cylinder repair at 1.2 MPa.

Figure 21 presents the hoop-stress distributions of the original steel cylinder within the repaired numerical configurations. The spatial variation around the prescribed broken-wire region reflects load transfer among the strengthening steel cylinder, grouting layer, and original pipe. The strengthening steel cylinder carries circumferential tensile force, while the grouting layer transfers radial pressure and coordinates deformation between the new and existing components. Quantitative changes between corresponding unrepaired and repaired configurations are evaluated from the strain–water-pressure curves in Figure 15, Figure 16, Figure 19 and Figure 20 and the reduction ratios in Figure 22.

For corresponding unrepaired and repaired numerical configurations with the same nominal wire breakage level and damage location, reduction ratios were calculated at 1.2 MPa from the hoop strains of the concrete core and original steel cylinder, as shown in Figure 22. The calculated concrete-core strain reduction ranges from 79.8% to 95.3%, and the calculated original steel-cylinder strain reduction ranges from 77.2% to 91.0%.

### 4.3. Response Characteristics of the Strengthening System Under Different Broken-Wire Conditions

The strengthening steel cylinder and micro-expansive concrete grouting layer are the key components in the repair system that participate in load redistribution and deformation coordination. To analyze the working state of the strengthening system under different wire breakage ratios and damage locations, the hoop strain of the strengthening cylinder, radial stress of the grouting layer, and hoop stress distribution of the strengthening cylinder were extracted and compared.

**Figure 23:**
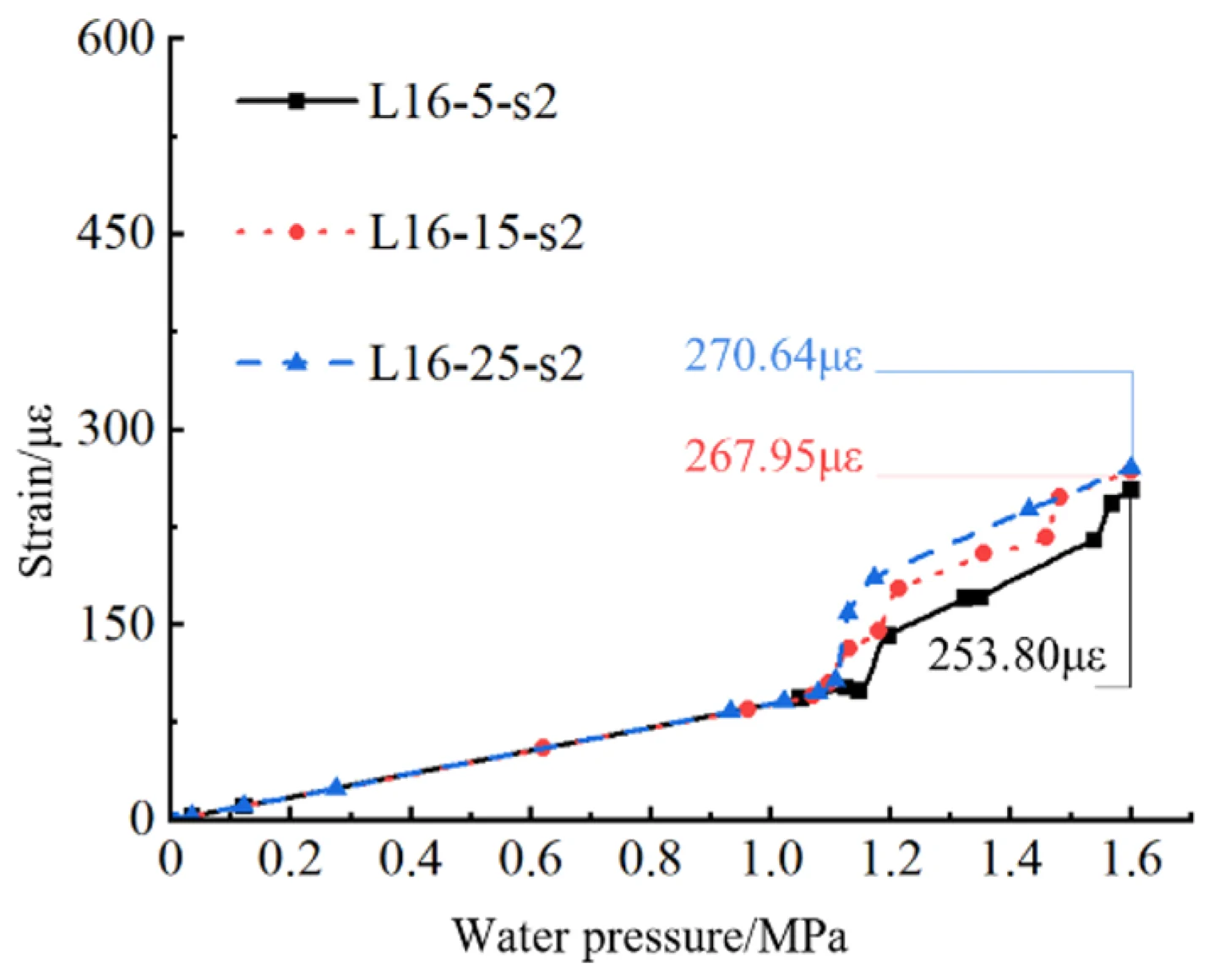
Strain–water pressure curves of the strengthening steel cylinder at the pipe-end socket region after strengthening.

**Figure 24:**
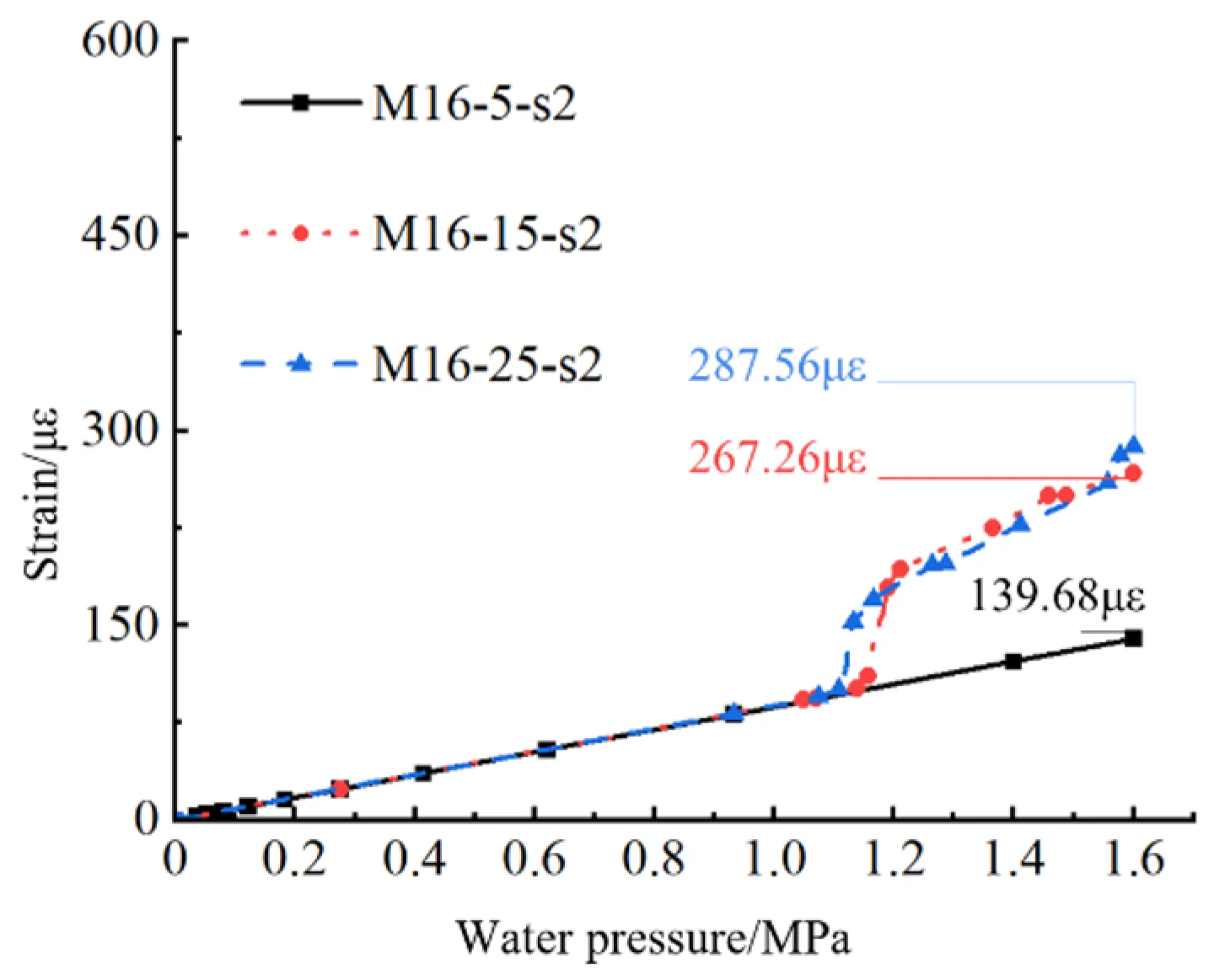
Strain–water pressure curves of the strengthening steel cylinder at the pipe midspan after strengthening.

Figure 23 and Figure 24 show that the strain of the strengthening steel cylinder increases gradually with water pressure during loading. The curves increase slowly in the early loading stage. When water pressure approaches the core-cracking stage, the strain growth rate increases, indicating that after local stiffness degradation of the original pipe, more hoop load begins to be borne by the strengthening cylinder. At the pipe-end socket region, the strengthening-cylinder strains at 1.6 MPa are 253.80, 267.95, and 270.64 με for 5%, 15%, and 25% broken-wire conditions, respectively. At the pipe midspan, the corresponding strains are 139.68, 267.26, and 287.56 με. These results indicate that increasing wire breakage ratio increases the calculated hoop tensile demand carried by the strengthening cylinder, while its modeled strain remains below 288 με over the investigated pressure range up to 1.6 MPa.

From the viewpoint of location differences, the strengthening-cylinder strain in the pipe-end socket region is higher than that at the pipe midspan under low wire breakage ratios, reflecting that end boundary effects still influence the local response of the repair system. Under 15% and 25% wire breakage ratios, the strengthening-cylinder responses at the two locations gradually approach each other. This indicates that as damage severity increases, the strengthening cylinder shares more local load and reduces response differences between damage locations.

**Figure 25:**
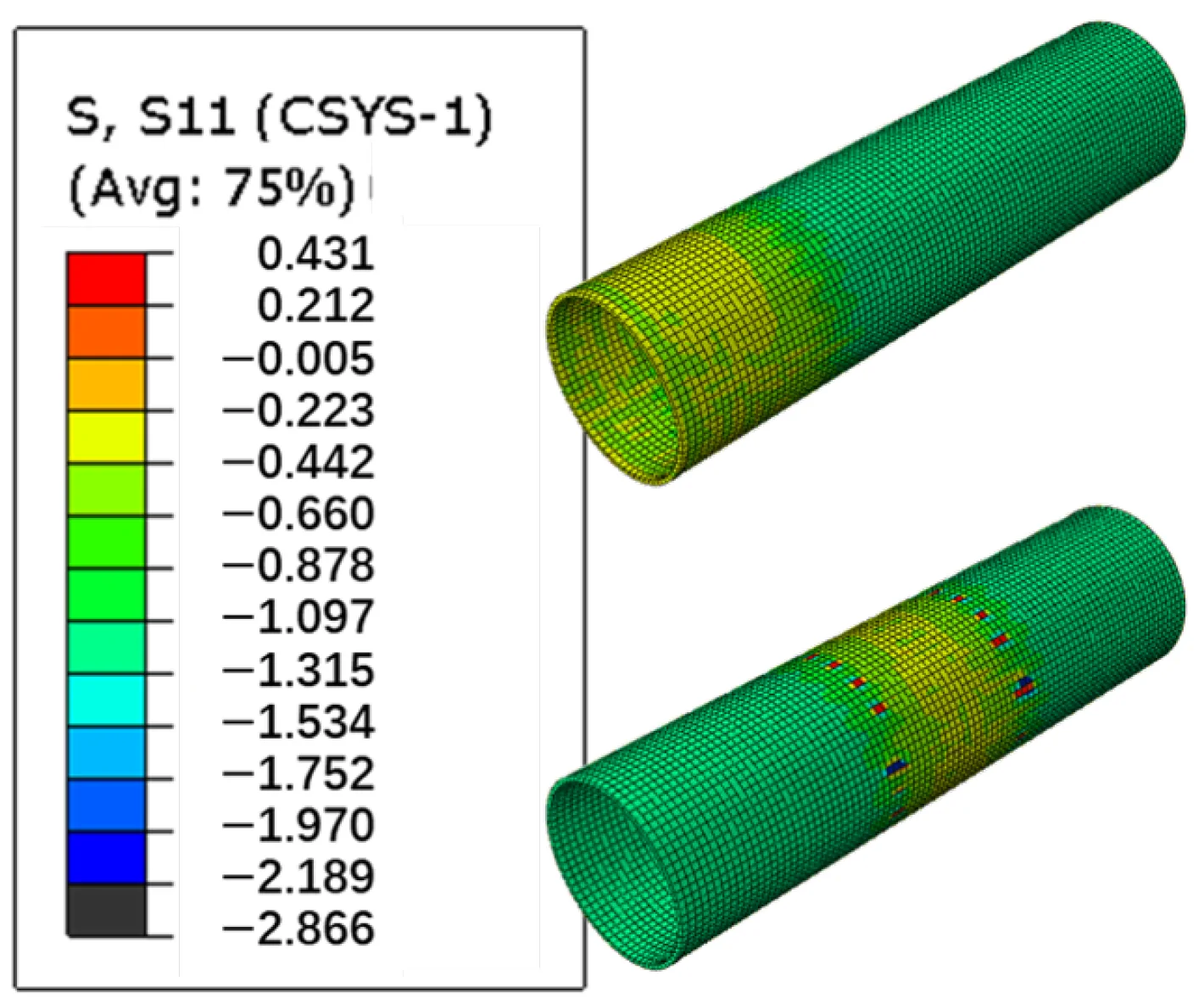
Radial stress distribution contours of the micro-expansive concrete layer at 1.6 MPa with a 25% wire breakage ratio.

The radial load-transfer function of the grouting layer is further illustrated in Figure 25, where the 25% wire breakage ratio is selected as a representative severe-damage case. At a water pressure of 1.6 MPa, the micro-expansive concrete grouting layer is generally in a radial compression state, showing that it forms a continuous compression-transfer medium between the strengthening cylinder and the original pipe core. Although radial stress fluctuates near the local broken-wire region, the overall state remains compressive.

**Figure 26:**
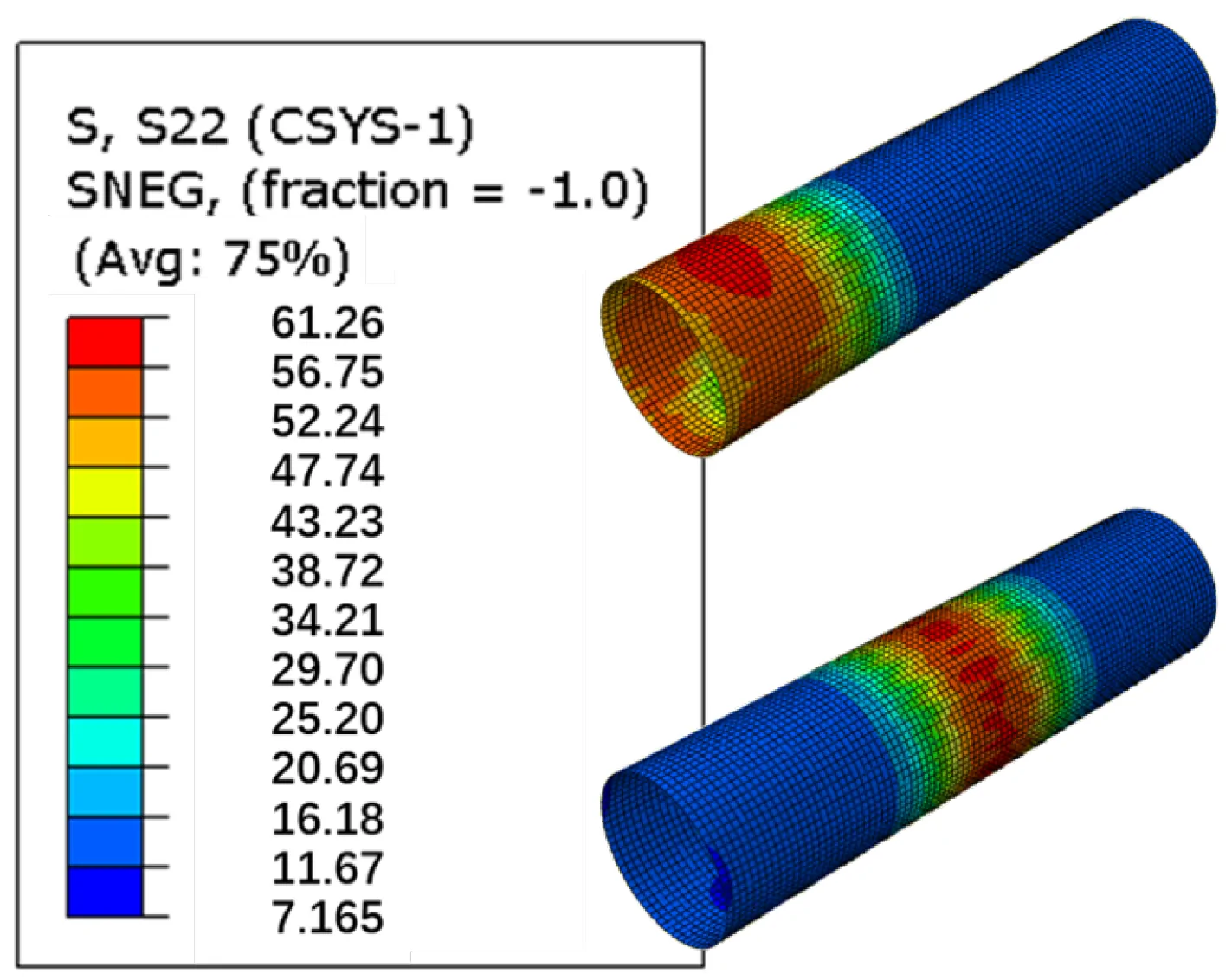
Hoop stress distribution contours of the strengthening steel cylinder at 1.6 MPa with a 25% wire breakage ratio.

Figure 26 further shows the hoop-stress distribution of the strengthening cylinder in the representative 25% condition. Local high hoop stress appears near the broken-wire region, but the high-stress zone does not develop through the entire structure. Under the pipe-midspan condition, the high-stress region is mainly concentrated near the damage window and gradually attenuates outward. Under the pipe-end socket condition, end boundary effects produce a more localized ring-like concentration.

Within the investigated monotonic internal-pressure range, the results shown in Figure 23, Figure 24, Figure 25 and Figure 26 indicate that the repaired system reduces the calculated strain demand of the original pipe and redistributes hoop and radial loads among the strengthening steel cylinder, grouting layer, and original pipe.

FRP/CFRP linings have been used to supplement the circumferential restraint of damaged PCCP [19,21]. External prestressed steel strands and prestressed CFRP provide circumferential strengthening from outside the pipe [24,25], whereas steel relining introduces an internal steel load-bearing layer [26,27]. In the present repair configuration, the component-level results show that the strengthening steel cylinder carries hoop tension, the grouting layer transfers radial compression, and the original pipe remains a participating component under monotonic internal pressure.

In engineering practice, the strengthening-cylinder dimensions and wall thickness should be determined according to the existing pipe geometry, damage condition, and operating pressure. Internal access and construction clearance are required, and the reduction in hydraulic area after lining should be checked against the required conveyance capacity. Construction quality control should focus on end connections, weld sealing, and complete filling of the annular grouting space. Post-repair inspection should examine leakage, local deformation, and grout continuity. These considerations are consistent with the principal implementation requirements reported for steel relining of large-diameter PCCP [26,27].

## 5. Conclusions

This study investigated the damage evolution and mechanical-response degradation of prestressed concrete cylinder pipes (PCCP) under multiple broken-wire conditions. Based on a three-dimensional finite element model calibrated by a full-scale hydrostatic-pressure test, test-calibrated parametric simulations were conducted for different wire breakage ratios and damage locations, and the performance of the internal steel-cylinder–micro-expansive-concrete grouting repair system was evaluated. The main conclusions are as follows.

1. Damage evolution in unrepaired PCCP is strongly affected by damage location. Owing to geometric discontinuity and boundary constraints, the pipe-end socket region has higher cracking sensitivity and stronger axial damage propagation than the standard pipe-midspan section. As the nominal wire breakage level increases from 5% to 15% and 25%, circumferential restraint is progressively weakened, the numerical nonlinear-transition pressure generally decreases, and the damaged zone expands from the broken-wire window to adjacent regions.
2. Broken-wire damage increases the hoop strain of the original steel cylinder after concrete-core cracking. At the pipe-end socket region, the original steel-cylinder strains reach 1822.02, 1902.50, and 2684.20 με under the 5%, 15%, and 25% configurations, respectively. The fixed-position response at the pipe midspan is locally non-monotonic between the 15% and 25% configurations and is interpreted as a response at the selected output position.
3. For corresponding unrepaired and repaired numerical configurations at 1.2 MPa, the calculated concrete-core strain reduction ranges from 79.8% to 95.3%, and the calculated original steel-cylinder strain reduction ranges from 77.2% to 91.0%. These values quantify differences under monotonic internal pressure, dense grouting, and tied interfaces. At the maximum investigated pressure of 1.6 MPa, the modeled maximum original steel-cylinder strain was 459.11 με, below the nominal yield-strain reference.
4. Under monotonic internal pressure up to 1.6 MPa, the repaired numerical configurations exhibit coordinated load sharing: the strengthening steel cylinder carries hoop tension, the micro-expansive concrete grouting layer transfers radial compression, and the original pipe remains a participating component. The full-scale loading program ended at 1.6 MPa without an ultimate-failure stage.

Future research will extend the present experimental–numerical framework to comparative assessment of internal and external repair configurations, combined internal-pressure and soil–traffic loading, cyclic and transient hydraulic actions, and long-term structural response associated with corrosion, prestress relaxation, and interface durability. Full-scale testing and long-term monitoring will be used to evaluate these repair systems under representative service conditions. 

## Figures and Tables

**Table 1:** Elastic properties of the unified numerical baseline.

Component and Model Material	*E* (MPa)	Density (kg/m^3^)	ν
Pipe-core concrete, C50	34,500	2500	0.20
Grouting layer, C60	35,992	2500	0.20
Mortar coating, M45	24,165	1850	0.20
Original steel cylinder, Q235	206,000	7850	0.30
Strengthening steel cylinder, Q345	206,000	7850	0.28
Prestressing wire	193,050	7850	0.30

**Table 2:** Concrete Damaged Plasticity parameters used in the numerical model.

ψ	ϵ	$f_{b0}/f_{c0}$	$K_{c}$	μ
$30^{\circ }$	0.10	1.333	0.6667	0.001

**Table 3:** Mesh-sensitivity results for the unrepaired 25% pipe-midspan configuration at approximately 1.2 MPa.

Mesh	Nodes	Elements	Core Mean (με)	Cylinder Mean (με)	$D_{t}>0.1$	(ALLAE+ALLSD)/ALLIE
C	25,446	19,196	723.1	725.7	71.28%	0.089%
M	50,438	38,310	723.8	723.8	71.81%	0.128%
F	117,564	93,224	724.6	706.6	71.43%	0.124%

## Data Availability

The raw data supporting the conclusions of this article will be made available by the authors on request.
